# Qbnb: An innovative C2B2C2B2C e-commerce mode for integrated on-demand services

**DOI:** 10.1371/journal.pone.0297593

**Published:** 2024-03-28

**Authors:** Yaoyao Wei, Cuiyin yao, Wei-fan Chen, Tian Xie, Jinzhao Yang

**Affiliations:** 1 School of Economics Management and Law at the University of South China, Hengyang, Hunan, China; 2 Information Sciences and Technology at The Pennsylvania State University, State College, Pennsylvania, United States of America; Wuhan Textile University, CHINA

## Abstract

The traditional supply-side-driven e-commerce modes provide consumers with massive amounts of structured information about goods and services. Due to the lack of a tailor-made ability to describe, explain, and accurately understand unstructured service demands, existing technologies and service modes still struggle to fully explore, articulate, and meet the personalized, high-quality, and urgent service demands of the disadvantaged groups in e-commerce. This paper innovatively proposes the concept of "integrated services on demand" and develops its C2B2C2B2C-based "Qbnb" e-commerce mode, operation mechanism and intermediary platform architecture. After conducting exploratory operation training, it demonstrates that the Qbnb mode can effectively achieve centralized allocation of idle social surplus service capacity to cater to the needs of disadvantaged groups in e-commerce. Compared to the traditional e-commerce service mode, Qbnb will significantly expand service demand and capacity, achieve higher service efficiency, and create a broader service consumption market, resulting in many more job opportunities.

## 1. Introduction

In the era of network information, eBay, Taobao, Uber, and other e-commerce platforms have significantly contributed to the creation and fulfillment of people’s structured demand for products and services. These platforms are empowered by the latest generation of ICT technologies, such as the Internet, the Internet of Things (IoT), big data, artificial intelligence, machine learning, and information push, among others. Technology is changing retail and customer service in various ways [1]. However, can the current technologies and service modes adequately extract, describe, and address people’s personalized, high-quality, and urgent unstructured service demands? Has machine intelligence surpassed human intelligence in this domain? The answer is evidently no, or it remains a topic of controversy. Even GTP-4 cannot comprehend people’s unstructured needs as efficiently and accurately as humans can.

### 1.1 Challenges of supply-side-driven

Whether it is Taobao, eBay, or any other platform, they are all supply-side driven in the way they present information about commodities on e-commerce platforms for customers on the demand-side to choose from. Only a few platforms offer personalized, high-quality products, and solutions from a demand-side perspective. Despite the support of artificial intelligence, machine learning, information recommendation, and other technologies, people are still dazzled by the enormous amount of available information [2]. Structured product and service information cannot accurately identify and match unstructured and ambiguous demand information, especially when customers do not have a clear understanding of their own needs. For example, when people encounter certain intractable diseases or problems, they can only describe their symptoms using vague language in their search for medical treatments or solutions Existing technology is consistently overwhelmed by such challenges, leading to extended periods where people are unable to swiftly and accurately obtain high-quality goods or services. In contrast, experienced medical professionals or even barefoot doctors can promptly provide precise understanding and diagnosis.

### 1.2 "Disadvantaged Group" and "Surplus Service Capacity" in the e-commerce environment

#### E-commerce disadvantaged group

The aging trend is increasing [3]. Elderly people find it difficult to operate mobile terminals and cannot easily use online shopping platforms to express and meet their service needs [4]. Meanwhile, for most young and middle-aged stable income earners, the high-intensity life rhythm and work pressure force them to deal with an increasing number of problems. They have less and less time and energy to actively search for personalized and high-quality products and services to satisfy themselves. In addition, for successful and well-funded social groups, the vast amount of information in the supply-side-driven e-commerce market has significantly increased the time cost of exploring their personalized and high-quality service demands, leading them to prefer paying for convenience and entrusting others to complete tasks. The aforementioned elderly, middle-aged, and young stable income earners, along with individuals with high time costs, have the financial means and service demands but lack the time or capacity, placing them among the e-commerce disadvantaged groups. As service demanders with the characteristics of "having demands and money" but "not having ability or time", they are defined as "Qer" in the Qbnb mode.

#### Surplus service capacity

There is another special group in the social network world corresponding to the e-commerce disadvantaged group. As the number of workers increases and the labor market gets younger, more and more online freelancers are popping up [5]. They do not have a stable income and lack sufficient capital, but they possess rich shopping experiences and the abilities to search for and select products and services quickly and accurately from the massively structured information in the supply-side-driven e-commerce markets. Most importantly, they have sufficient leisure time and learning ability to search for satisfactory solutions for personalized, high-quality, ambiguous, and unstructured product and service demands in a complex e-commerce network environment. We define this social group as the surplus service capacity in the e-commerce environment, mainly including students, jobless people, flexible entrepreneurs, and employed people, etc. Uber drivers are representative of this, with greater flexibility in labor supply and a corresponding surplus in service capacity [6]. As the service providers with the characteristics of "having time and ability" but "not having money" they are referred to as "Soer" in the Qbnb mode.

Whether it is product purchase agency or takeaway delivery, talent recruitment or confusion resolution, professionalism ensures the provision of high-quality services. In the existing supply-side-driven e-commerce platform environment, it is especially important to integrate social idle surplus service capacity and train professional e-commerce service virtual organizations to help e-commerce disadvantaged group to identify their actual demands and provide personalized, high-quality, and precise solutions. For companies, customer-centric business model innovation can also uncover higher product or service value [7].

### 1.3 The core concept “Integrated service on demand” for “Qbnb”

**Fig 1:**
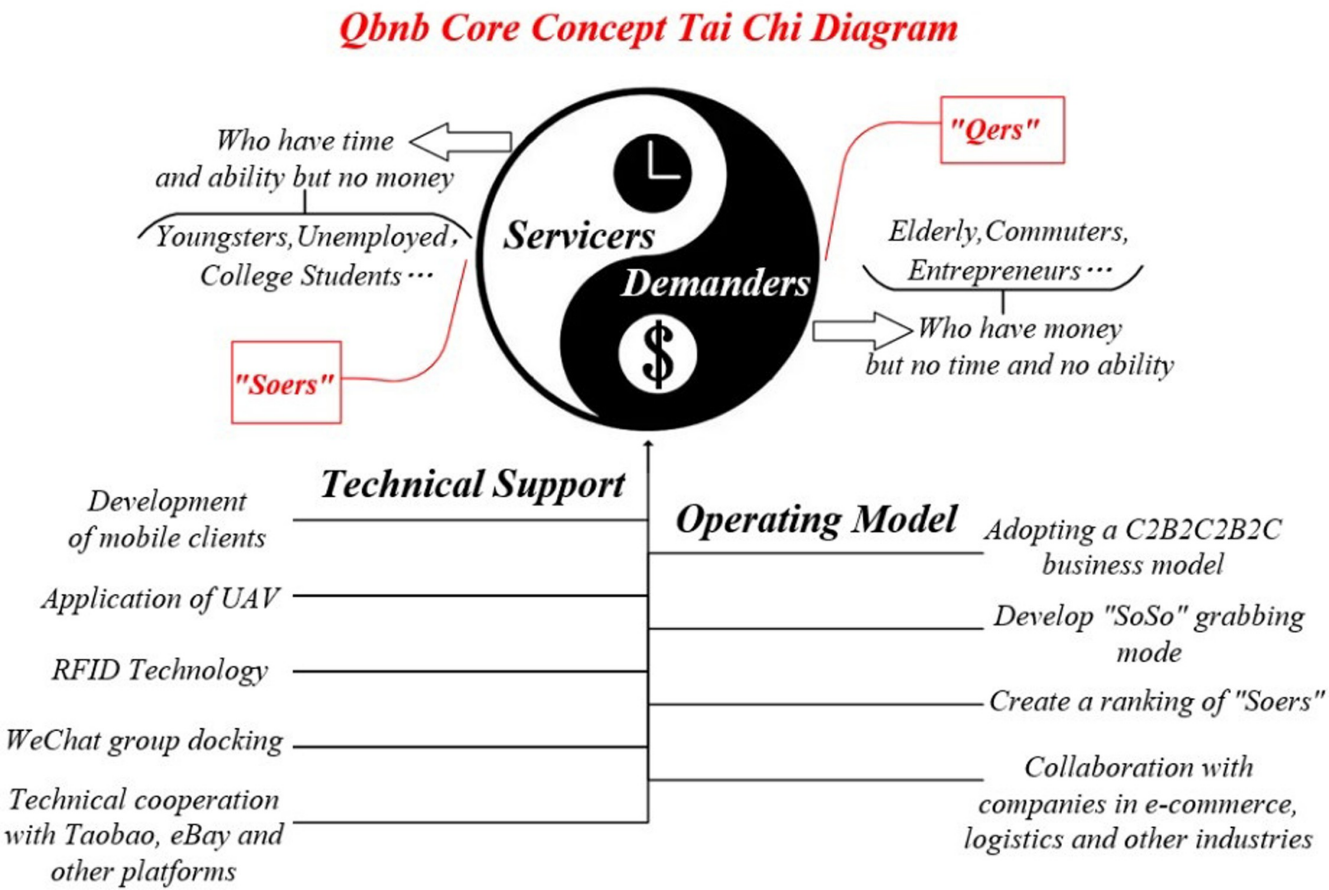
“Qbnb” core concept Tai Chi diagram.

Supply and demand in the e-commerce market are like the Yin (negative) and Yang (positive) sides of Tai Chi. Instead of opposing each other, they can complement, blend, and even transform one another [8]. Only when they are seamlessly integrated can the full potential value of the e-commerce market be stimulated and realized. Based on the existing business mode, this paper aims to explore the "Qbnb" on-demand integrated service mode, which follows the C2B2C2B2C approach, by researching a third-party intermediary platform that focuses on customer demands. The core concept of the Qbnb mode is represented by the Tai Chi symbol in Fig 1. In the Qbnb mode, both the e-commerce disadvantaged demanders (Qers) and the social surplus service providers (Soers) are considered as the client roles of intermediary platform. The service demand and payment ability of Qers, as well as the capital demand and service ability of Soers, can be fully integrated (merged, interacted with, and even transformed) within the intermediary platform. The idle "having money and demand" and "having time and ability" of the e-commerce market can be effectively integrated and reasonably matched in this mode, enabling the centralized allocation of Soers to serve Qers on demand.

The subsequent contents of this article are organized as follows: Section 2 reviews and analyzes the characteristics, advantages, and shortcomings of the current mainstream shopping platform’s e-commerce operation mode. Inspired by the travel service mode, Section 3 proposes the Qbnb integrated on-demand service mode, which includes the C2B2C2B2C e-commerce mode and its operation mechanism. Section 4 presents the model and architecture of the "Qbnb" intermediary platform, discussing the key supporting technologies required for the system based on the technical requirements of the operating mode. In Section 5, typical online and offline practice cases and trial operation data are analyzed through exploratory case studies to demonstrate the effectiveness and market prospects of the Qbnb mode. Furthermore, Section 6 discusses the advantages and values of Qbnb as a new business mode. Finally, Section 7 presents a conclusion and outlines future work.

## 2. Literature review

### 2.1 Taobao, eBay, Amazon, and other purchase platforms

**Fig 2:**
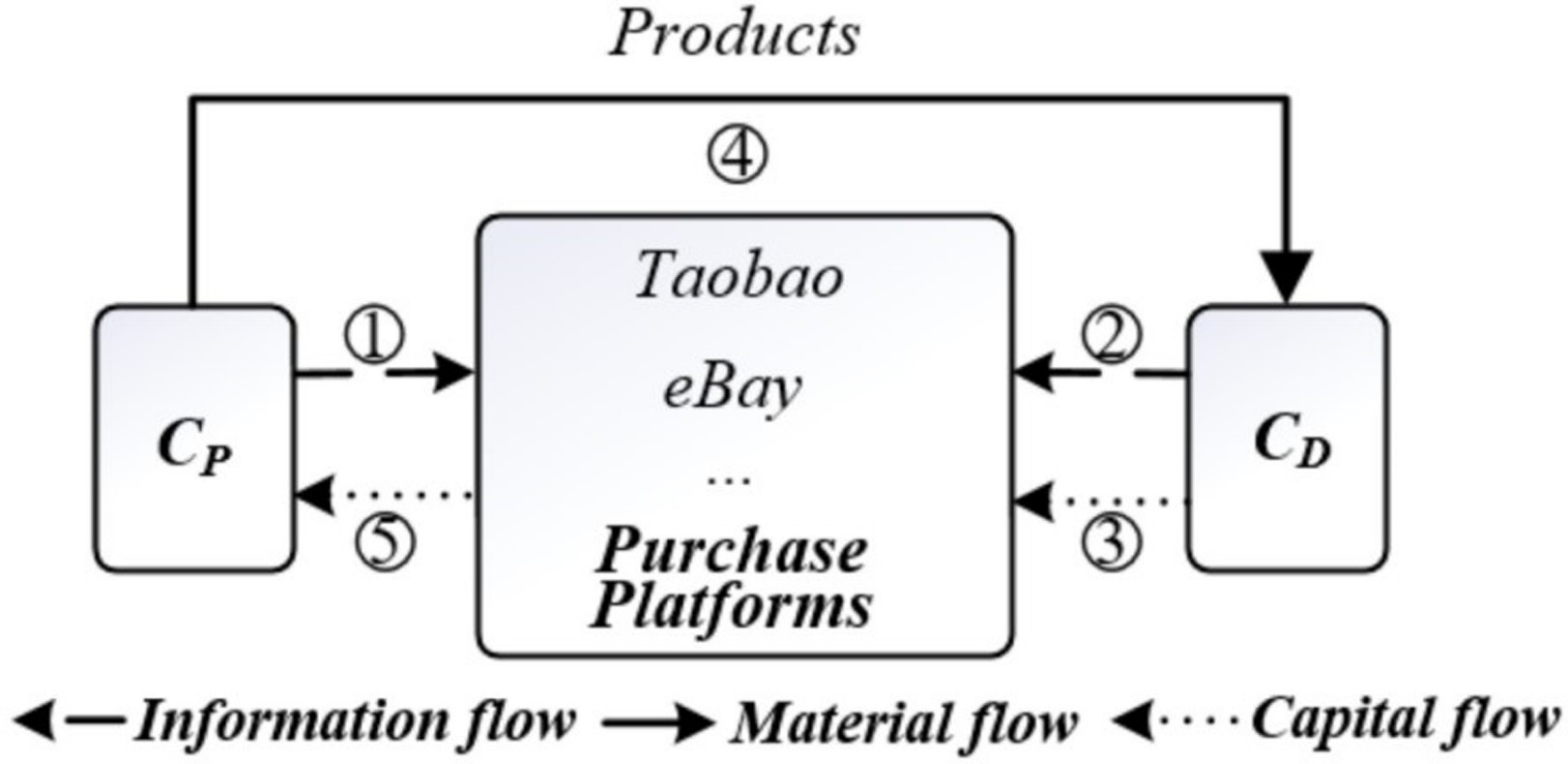
C2C e-commerce mode of purchase platforms such as Taobao and eBay.

For traditional C2C e-commerce purchase platforms such as Taobao and eBay, their customers include Product Providers (C_P_) and Product Demanders (C_D_). Due to the supply-side-driven attributes of these platforms, providers first publish the information of saleable goods in a specific structure on the platform, and demanders search the supply information on the platform using keywords to match their purchasing demands. Through communication and negotiation between them, prices are determined, and agreements are formed. Demanders pay the platform, and providers ship the goods to demanders through offline logistics. After confirming that the goods are correct, the platform will pay the providers. Finally, both parties evaluate each other and complete the transactions (Fig 2), which ensured the quality of goods and the security of funds to a certain extent.

As one of the largest online sales platforms, eBay offers online auction and retail shopping services to the global public [9]. However, eBay does not directly participate in the purchase and sale of goods; instead, it provides a platform for communication and transactions between buyers and sellers, making it a typical example of a global C2C e-commerce platform. Similarly, Taobao, the largest e-commerce platform in China, also provides a third-party trading platform for buyers and sellers. The difference is that Taobao incorporates more localized features in China [10]. Both eBay and Taobao serve as intermediaries between buyers and sellers, offering consumers a wider range of goods than offline merchants, and also providing more opportunities for self-employed business man and small/medium-sized enterprises, which play an important connecting role in the transactions [11].

The Amazon platform adopts a B2C business mode, providing automated tools for third-party sellers [12]. Amazon offers advanced servers that make it easier and faster for users to retrieve goods and also provide customers with a free preview function. However, due to its content limitations, it is challenging for consumers with unclear goals to decide what kind of goods to buy in a short time, which ultimately limits the enhanced shopping experience and customer satisfaction.

Alibaba, as one of the world’s largest B2B e-commerce platforms, focuses on building communication channels between enterprises and greatly facilitates trade between China and the rest of the world. The Alibaba e-commerce platform brings more significant opportunities for enterprises than traditional import/export trade models. However, for personalized retail purchasing consumers, the enterprise-oriented batch trading e-commerce platform may not be conducive to effectively obtaining a good service experience [13].

Taobao, eBay, Amazon, Alibaba, and other C2C, B2C, and B2B e-commerce modes have provided a large number of consumers with convenient third-party platforms for online shopping. However, from a supply-side perspective, these platforms provide a vast array of commodity information for the demand side to choose from. This is particularly true for Qers, a disadvantaged group of personalized e-commerce customers with high time costs or unclear demands, as they lack tailor-made e-commerce services. Their trading decisions are easily flooded and confused by "related" recommended commodity information, consuming a lot of time without satisfying their initial real demands. This shows the traditional business service model has found it difficult to meet the enterprise integration platform resources and create more customer value [14].

### 2.2 Inspiration from Didi, Uber and other service platforms

**Fig 3:**
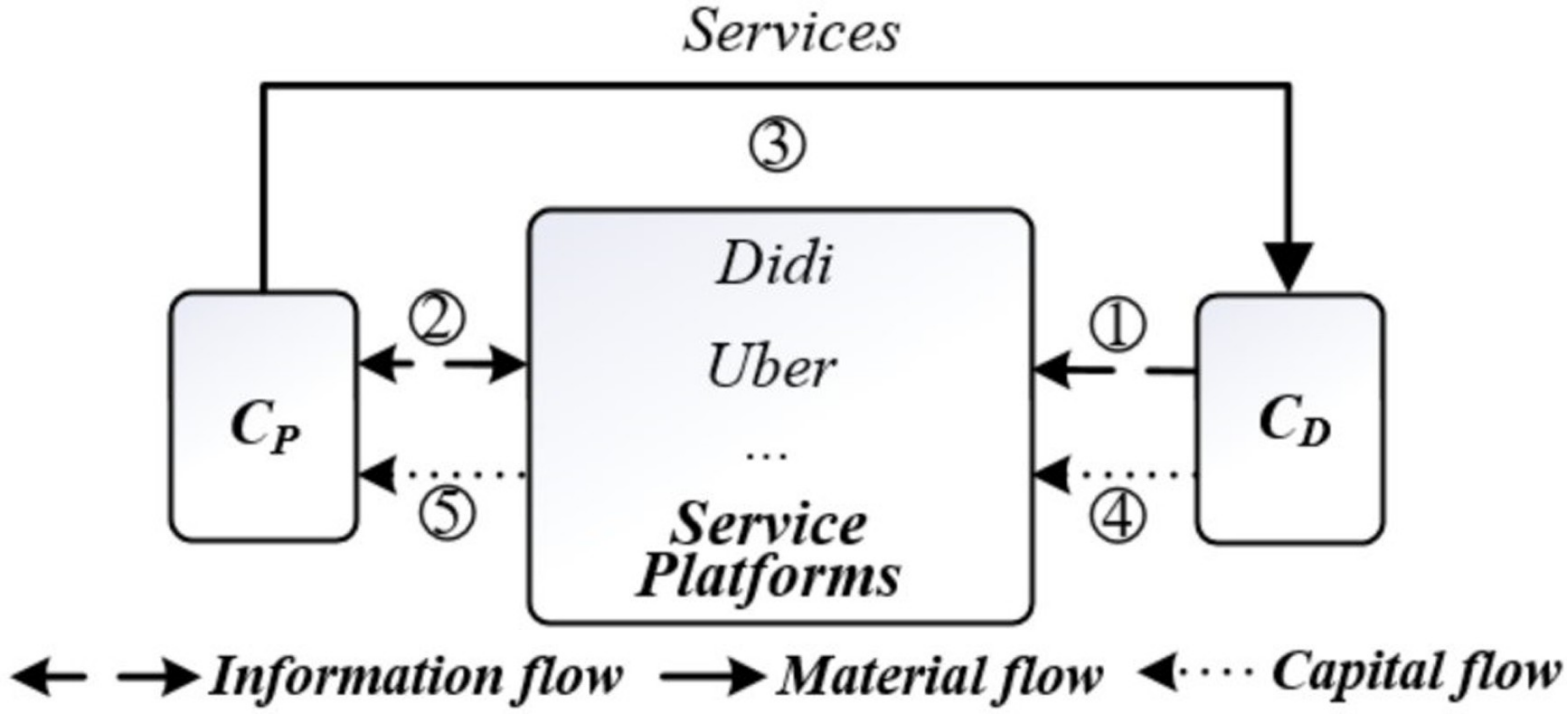
C2C e-commerce mode of service platforms such as Didi and Uber.

Similar to Taobao and eBay, the customers of Didi and Uber’s service platforms include Service Providers (drivers, C_P_) and Service Demanders (passengers, C_D_). First, the service demanders release demand information such as departure location, travel time, and target location on the platform. The platform then notifies the service providers near the departure location and matches supply and demand through "order grabbing" and "car pooling." The service providers offer ride services to the demanders. Upon reaching the destination, the demanders pay fees to the platform, which then deducts the intermediary fee and disburses the remaining amount to the service providers. Finally, both parties evaluate each other, completing the transaction (Fig 3).

Didi, Uber and other C2C service platforms use the mobile Internet to provide third-party integrated services, which subverts the traditional concept of "taxi." This enables drivers to "grab the orders" according to the passenger’s destination, reducing the empty driving rate and maximizing the resources and time of both drivers and passengers [15].

Distinct from supply-side-driven e-commerce platforms such as eBay, Amazon, and Taobao, the transaction behaviors of Uber and Didi are triggered by demand. Didi and Uber provide only taxi travel services, but these demand-triggered service transaction behaviors offer us a new idea to break away from the single supply-side driven C2C, B2C, and B2B e-commerce modes and explore a new demand-side driven diversified e-commerce service mode. We can design an integrated platform for third-party demand release and service response to effectively utilize idle surplus service capabilities and meet the personalized and high-quality demands of disadvantaged groups in the E-commerce environment.

## 3. “Qbnb” e-commerce mode for integrated service on demand based on C2B2C2B2C

### 3.1 The innovative “C2B2C2B2C e-commerce mode” for “Qbnb”

**Fig 4:**
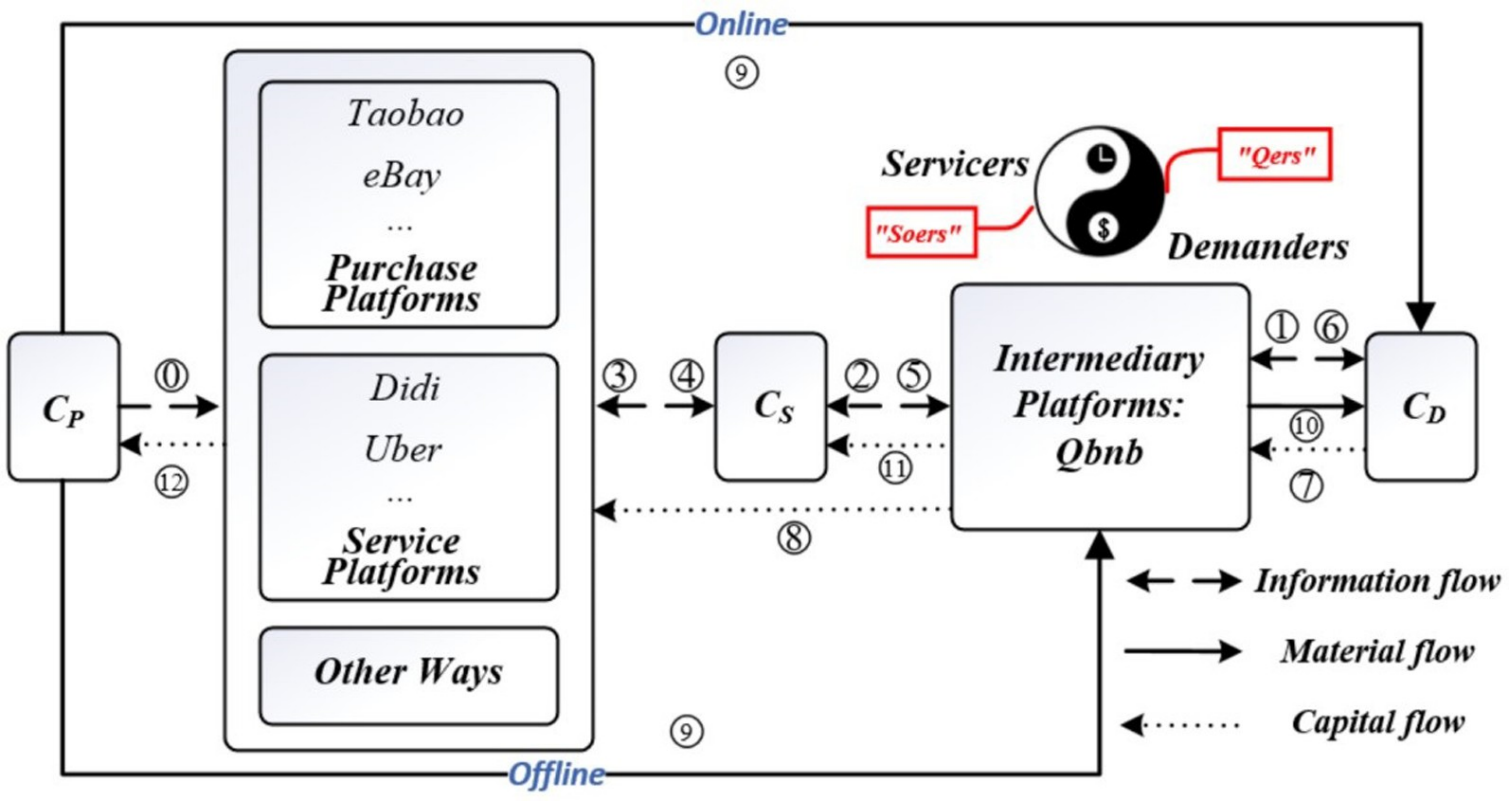
Business mode of C2B2C2B2C for third-party demand release and integrated service response platform.

We innovatively integrate the "Qbnb Intermediary of third-party demand-driven integrated services Platform" and the surplus service capability providers "Soers (C_S_ side)" into the existing provider-driven e-commerce mode, effectively combining the advantages of purchase platforms and service platforms. This integration occurs between the Purchase/Service Platforms and Demanders side (C_D_ side). The specific C2B2C2B2C-based e-commerce service mode is shown in Fig 4. Demanders ("Qers") on the C_D_ side send service requests (explicit or vague) to the intermediary platform called "Qbnb." The platform communicates, analyzes, and organizes the demands, translating them into orders to be published to a specialized group of “Soers” that provide the relevant types of services. Through "order grabbing" and "communication with Qers," the service providers (Soers) are verified, and a service agreement is established. The Qers will temporarily pay the agreed service fees to the "Qbnb" platform for safekeeping. As per the agreement, the Soers will fulfill the Qers’ demands by either making purchases on existing mainstream e-commerce platforms, opting for third-party services on the service e-commerce platform, or exploring other solutions. In the case of purchasing services, the C_S_ side’s Soers will replace the Qers as the demand side on eBay, Amazon, and other platforms. They will search for satisfactory products on various e-commerce platforms on behalf of the Qers who either lack time or do not possess e-commerce shopping capabilities. As a Soer specialized in dealing with a certain type of demand, he possesses rich experience and incurs a lower time cost, making him several times more efficient than Qers. Within the agreed time, Soers deliver the corresponding online or offline solutions to the platform. They rely on the platform’s customer service staff to communicate with Qers and obtain feedback (whether optimization or re-service is needed). The Qbnb platform will compensate Soers for their services, and both parties involved in the service will evaluate each other. The transaction is then considered complete.

Based on the "Qbnb" intermediary platform, the "Qbnb" mode integrates service providers ("Soers") with "time and ability" and service demanders ("Qers") with "demand and money." The "Qbnb" mode reduces the complexity of operations and potential communication barriers on the user side in the traditional C2C process. Instead, it delegates these issues to the platform and professional solution researchers (“Soers”) to cater to the high-quality and urgent demands of the e-commerce disadvantaged groups and other users. The "Qbnb" platform collects the payment from Qers in advance and then transfers it to the Soers and the traditional purchase or service e-commerce platforms after the service is completed. The traditional platforms, in turn, pass it back to the original suppliers of the products or services, ensuring the security of funds. In addition, the "Qbnb" platform can either directly purchase the commodities or send the commodity purchase links provided by the original suppliers (C_p_ side) to Qers, based on their specific demands. This approach enables the platform to achieve diversified search and delivery of both online and offline, tangible and intangible services, ensuring efficiency and a hassle-free experience for users.

### 3.2 “Qbnb” mode of operation mechanism

**Fig 5:**
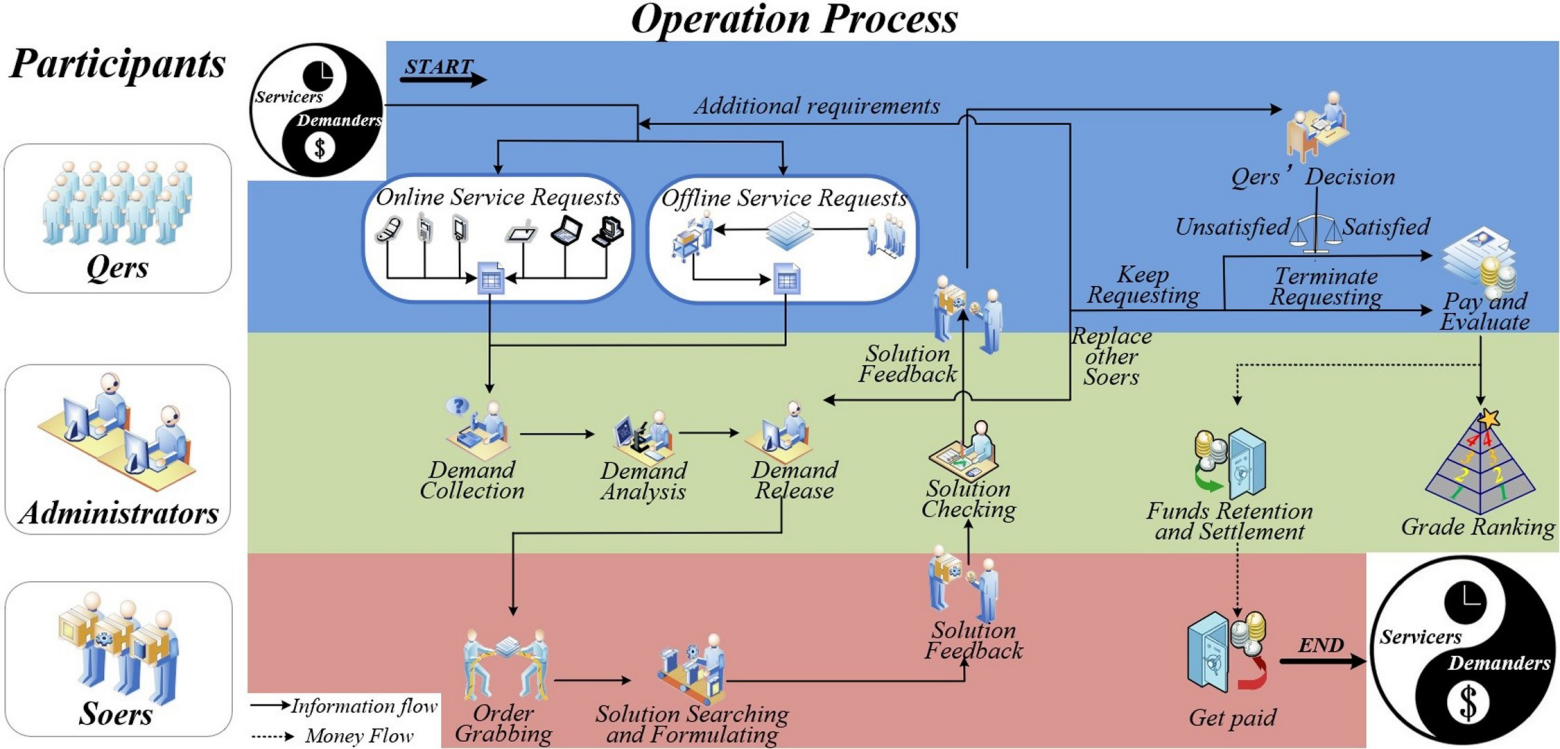
“Qbnb” operation process.

With the essential technical support of Qbnb intermediary platform and E-commerce logistics, we have developed an online-offline parallel operation mechanism for Qbnb from the perspective of three types of users: "Qers, Administrators, and Soers (Fig 5)."

#### "Demand processing”

**Fig 6:**
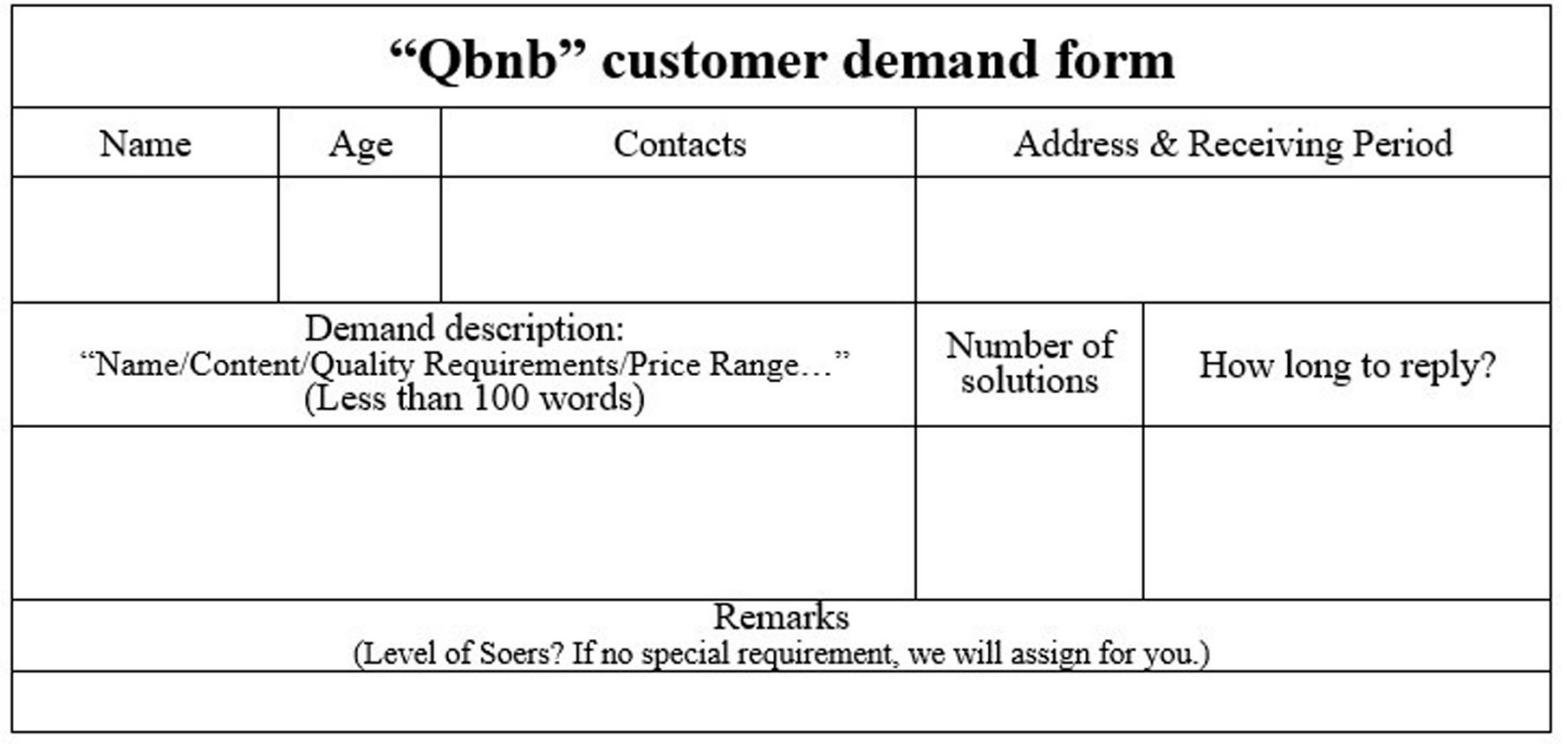
The “Qbnb” electronic demand form.

**Fig 7:**
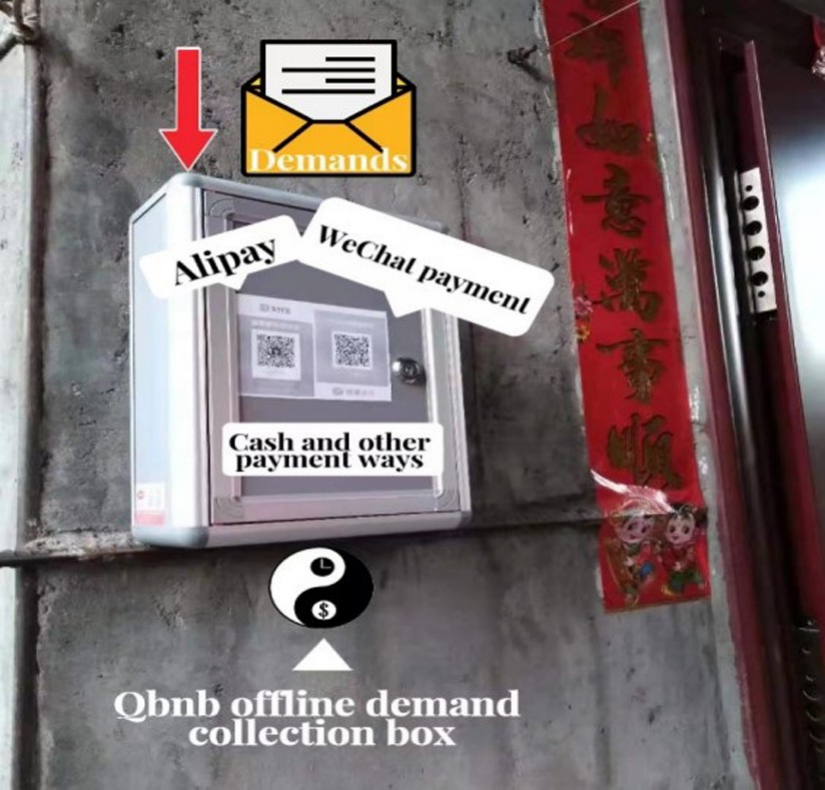
“Qbnb” boxes for collecting demands.

**Fig 8:**
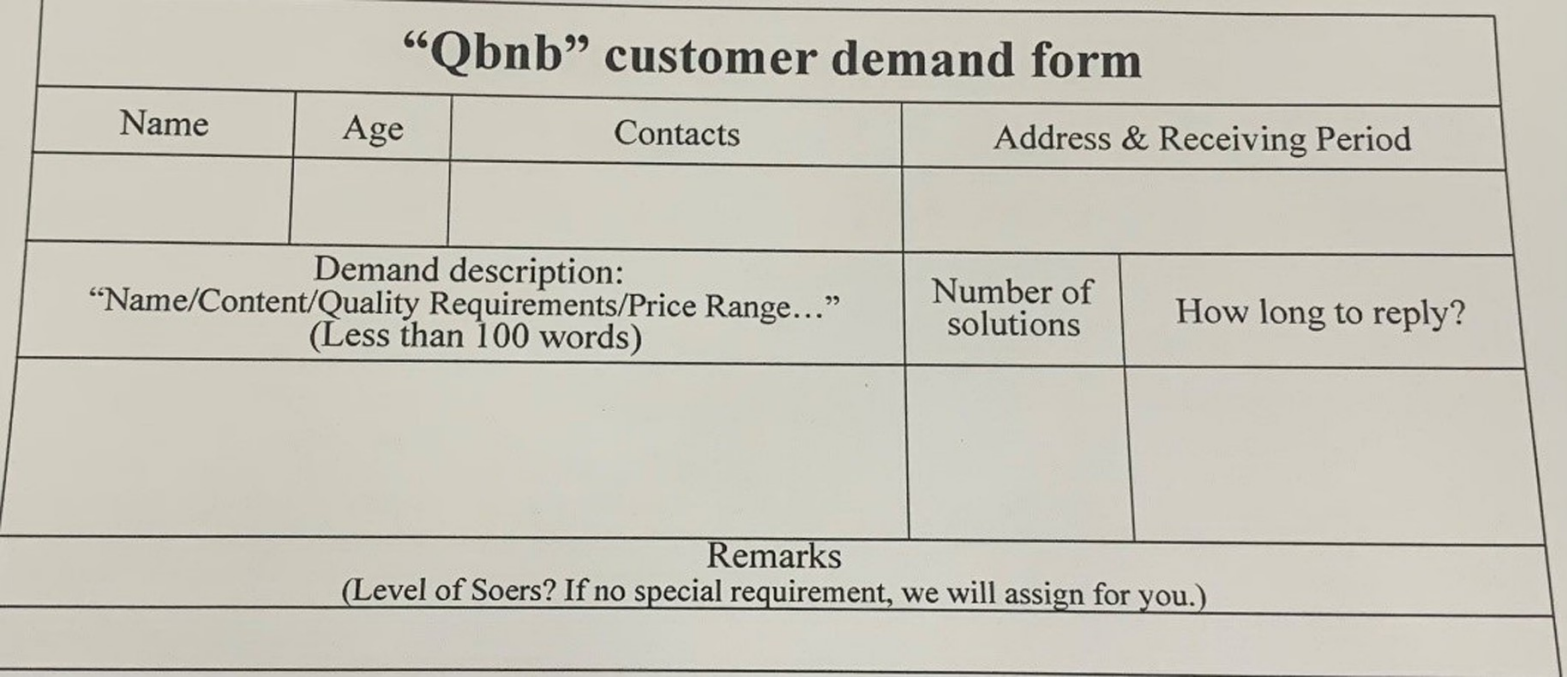
“Qbnb” paper service demand form (translated).

"Demand processing" includes three steps: demand collection, demand analysis, and order release. During the demand collection step, Qers can make unstructured (or even vague) service requests to the Qbnb platform from both online and offline channels. For Qers who frequently use smartphones, computers, and other (mobile) Internet terminals, they can transmit their demands to the platform’s administrators by filling out electronic demand forms (refer to Fig 6) or sending voice demand messages. For elderly Qers who are not proficient in using Internet terminals, each residence is equipped with a "Qbnb" mailbox (Fig 7) intended for receiving paper service demand forms (Fig 8) and prepaid cash service fees (QR codes for Alipay and WeChat payment are attached to the mailbox’s surface). The collected demands are then stored and analyzed by administrators. Subsequently, depending on the type of demand, it is transformed into an order and released to Soers group, which specializes in handling such service demands to provide a more efficient on-demand service.

#### (2) “Order Grabbing"

The Qbnb platform classifies, evaluates, and manages orders and Soers by establishing separate order information databases and Soers information databases respectively. Through the creation of a "grab pool," it enables the ranking and recommendation of Soers willing to accept orders based on Qers’ preferences.

**Fig 9:**
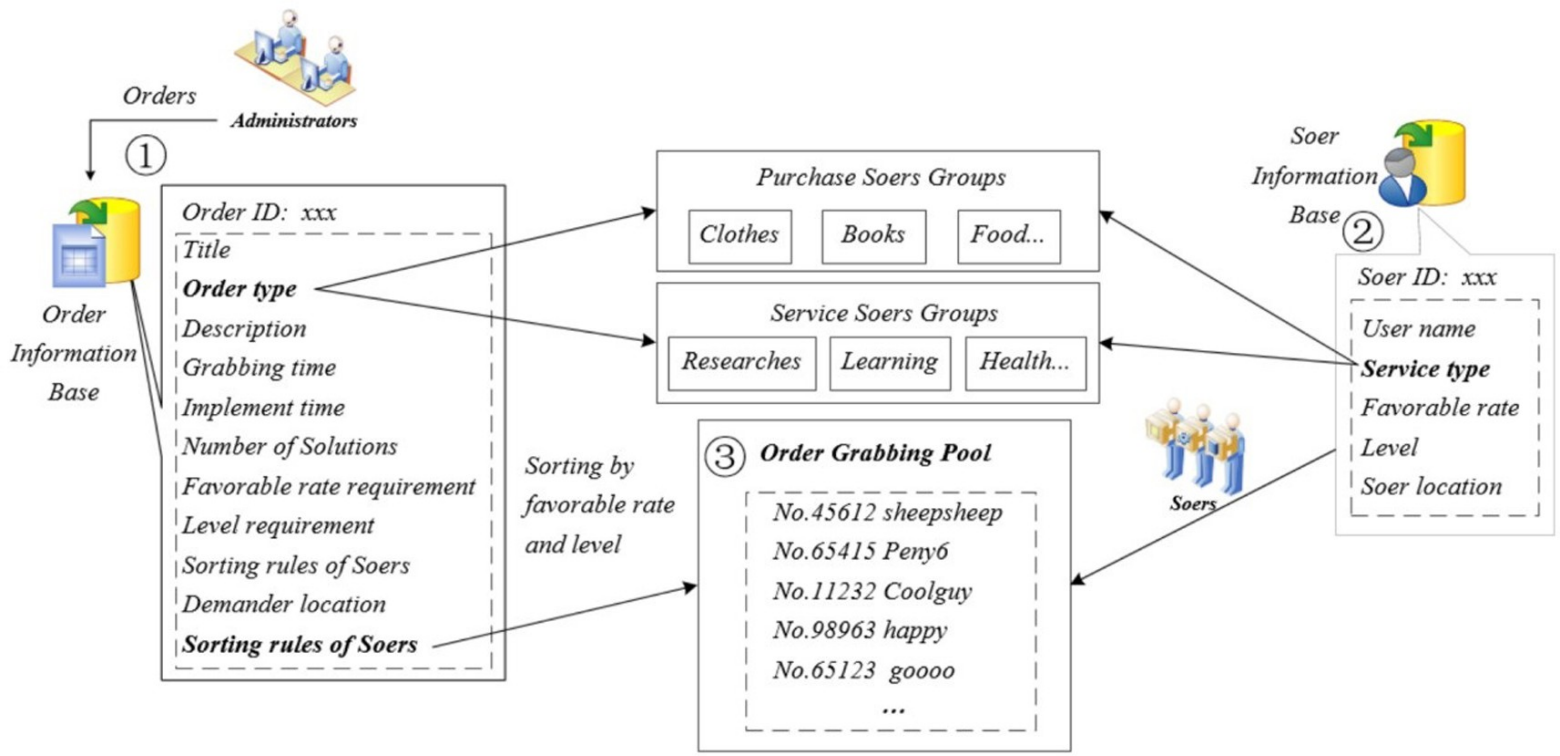
“Qbnb” order-grabbing process.

As shown in Fig 9-②, each Soer has its own key attribute values, including ID, Name, Service Type, Favorable Rate, Level, Soer Location, et al. The Service Type identifies the type of service the Soer is proficient at providing and which Soer Group it belongs to on the Qbnb platform. The Favorable Rate is an evaluating indicator of the quality of a Soer’s service after they have provided many services. Level is the grade of each Soer on the platform, and it is the weighted sum index of their service quality, service quantity, contribution, reputation, and experience. The rules for calculating the Favorable Rate and Level are presented in the "Pay & Evaluation" section.

As shown in Fig 9-①, the order information base contains key attribute values for each order, including ID, Title, Order Type, Description, Grabbing Time, Implement Time, Number of Solutions, Favorable Rate Requirement, Level Requirement, Sorting Rules of Soers, Demander Location, and more. The Order Type identifies the nature of the order, such as a purchase of goods, a query to be resolved, or a search for medical treatments for intractable diseases. Description is a specific, unstructured representation of the Qers’ demands that contains important information about the desired goods or services, such as specifications, functions, models, willingness to pay prices and service fees, etc. Grabbing time is the duration set by Qers to accept orders after a specific Soer group posts an order, and Soers can only accept orders within this time interval. The Favorable Rate Requirement and Level Requirement represent the minimum criteria that Qers need to meet for the favorable rate and level attributes of the Soers when accepting orders, respectively. The sorting rules of Soers are the strategies employed by Qers in the order-grabbing pool to select Soers. These rules can be determined based on the level or favorable rate of Soers. Alternatively, they can involve multi-objective sorting, using the primary criterion of favorable rate and the secondary criterion of level. We can even develop an optimal algorithm to achieve this function by drawing inspiration from the "order grabbing" model used by some takeovers and taxi drivers [16–18].

The specific order-grabbing process is shown in Fig 9. Administrators will post the order to the corresponding Soer (sub-)group according to the order type. The Soers in the group who are willing to take the order will choose to do so. Within the limited grabbing time, the Soers who press the “Take Order” button or send the "willing to take order" message will be listed in the order-grabbing pool. According to the "Sorting rules of Soers" provided by the order, the Soers who are in the top 5 or 10 positions will be ranked and listed for the Qers to choose from. Finally, based on the “Number of Solutions” requirement in the order, Qers can select one or more Soers to fulfill the order and provide services.

#### (3) “Solution searching, formulating, feedback & check”

After grabbing the order, Soers accurately interpret Qers’ unstructured and ambiguous service demands, taking into account the specific requirements and descriptions of the order. They utilize their own professional experience, fixed channels, third-party e-commerce platforms and other resources to search for solutions or services that can satisfy Qers on the Internet or in reality. According to the specific requirements of Orders, Sers can not only provide solutions but also offer complete problem-solving behavior on demand. For example, for some online purchase orders, Soers can either provide a satisfactory online purchase link or directly purchase and send the goods home according to Qers’ demands. The solution will be sent back in the form of a document to the platform administrator for review, with special emphasis placed on the confidentiality of Soers and Qers’ personal information. This is to prevent Soers from contacting Qers privately and engaging in other illegal operations, ensuring the privacy of both parties and protecting the interests of the Qbnb platform. Section 5, "Exploratory Case Studies," provides specific details regarding this section.

#### (4) “Qers’ decision-making & pay”

The approved solutions are sent to Qers for a final decision after review by the administrators. If the Qers are not satisfied with the solution, they can choose to ask the original Soers to continue the service and find a new solution. Alternatively, they can go back to the initial stage to modify the demands and release the orders, and the Soers will re-grab the order to complete the service, or they can terminate the service request after paying the platform and the original Soers a basic fee. If the Qers are satisfied with the solution, they can proceed to pay the relevant fees and evaluate the services and Soers.

Qbnb follows the principles for service tips,which involve firstly, abiding by market orders and voluntary transactions. Qers estimate and quote the required tips for services when they submit information about their demands. If the service cost is too high or too low, especially too low, it becomes difficult to motivate Soers to grab orders to perform the service. During this process, the platform matches similar (historical) service pricing information to Qers for reference, based on the type of service (e.g., for product purchase services, the tips can range from 10% to 15% of the price of similar products). Secondly, the higher the amount of service involved, the lower the service tip ratio. Thirdly, the more difficult the service, the higher the tip ratio. Fourthly, the higher the level of Soers, the higher the tip extraction ratio. Fifthly, payment is made first, and then the service is provided. Qers pay the entire service transaction amount, including tips and service costs or commodity purchase costs, to the Qbnb platform for temporary storage when they raise their demands. Then the platform transfers the tips to the corresponding Soers after the services are completed, and Soers will advance the service cost or commodity purchase cost during the period. Sixthly, the platform management fees can take up to one-third to one-half of each trade’s tips.

#### (5) Mutual evaluation between Qers and Soers

Soers and Qers evaluate each other after service transactions. The Qbnb platform adopts a two-standard mutual evaluation system that considers the attributes of Level or Favorable Rate. The single favorable rating indicates how much the Qer/Soer approves of the Soer/Qer after single service, while the Favorable Rate is the average of mutual ratings after multiple service transactions. The Level attribute is used to identify the degree of excellence of a Soer or Qer over time. Assume that “*△Experience = α·Favorable Rate + β·Transaction amount / maximum transaction amount of similar services + γ·Service difficulty factor (number of service failures)/ maximum difficulty factor of similar services + δ…*” Experience is the cumulative value resulting from the weighted increment of the system’s comprehensive evaluation of service quality, quantity, amount, service difficulty, and service value of a Soer or Qer after each transaction. The cumulative value of the metric Level is “*Experience = ∑△Experience*”. We rank the top 1% of Soers or Qers in terms of Experience value as the highest “S” level (most excellent) on the leaderboard. The first 2–5% is ranked as A level, 6%-20% as B level, 21%-50% as C level, and 51%-100% as D level. Soers’ service fees will be linked to their levels, meaning that the higher the level, the higher the commission.

The Level-based evaluation system is the platform’s first choice because it comprehensively considers various factors. However, relying solely on this system is extremely unfair to the new Soers, as they may not have enough service quantity and transaction amount, resulting in a low Experience value. This situation is not conducive to their selection by Qers. Therefore, the platform has established a "training period" during which Qers or Soers can be evaluated and selected based on the Favorable Rate criteria. After the training period, the mainstream Level assessment system takes effect. This long-term, dynamic ranking system applies to both Soers and Qers, effectively motivating both parties to enhance the quality and efficiency of their services.

## 4. “Qbnb” platform technical support and architecture

### 4.1 Technical support

As shown in Fig 1, the Tai Chi diagram illustrates the comprehensive application of new ICT technologies such as Unmanned Aerial Vehicle (UAV), RFID, access and integration of third-party platform resources, mobile communication social technologies. This combination can provide a relatively complete platform solution for the Qbnb mode of on-demand integrated services in terms of technical implementation.

#### 4.1.1 Application of mobile terminals

Today, thanks to the development of social networking technology, individuals can now post their demands for products and services online using smart mobile devices [19, 20]. In the Qbnb mode, mobile terminals play a crucial role, becoming an important means for Qers to express their demands and seek services. According to the demands put forward by Qers, Soers can easily utilize mobile terminal devices to access vast service information and quickly find corresponding solutions on third-party e-commerce platforms. By developing an APP or an applet, a prominent demand application button is placed for Qers on the front-end page of the mobile client, aiding them in conveniently expressing their own demands. Corresponding function pages for Soers, such as order management and receipts, can also be established to address the demands put forward by Qers promptly.

#### 4.1.2 Application of WeChat and WhatsApp

As an instant messaging app, WeChat’s number of users has grown exponentially since its launch. Today, it has more than 1.2 billion users worldwide [21]. WeChat provides a good publicity platform and payment method for users on both sides of the transaction [22], making it easier to promote the success rate of commodity transactions. WhatsApp, similar to WeChat, is another global instant messaging tool. WhatsApp is considered to provide a relatively higher level of behavioral privacy, and it is also mainly used to interact with close contacts [23], making it more conducive to intimate communication between buyers and sellers.

Section 5 shows how we can achieve exploratory operational training with the technical support of WeChat. Due to WeChat’s advantages of "comprehensive functions, many users and social convenience," both WeChat and WhatsApp are expected to emerge as mainstream technologies for realizing the Qbnb mode, regardless of whether the Qbnb platform is developed and widely applied. The seamless integration between WeChat, WhatsApp, and the Qbnb platform will also be a key area of research in the future.

#### 4.1.3 Application of UAV

In addition to the online service demands collected by mobile terminals, the Qbnb platform also takes into account the offline service demands of disadvantaged groups who may not be proficient in using mobile terminals and the Internet. In the post-pandemic era, door-to-door collection of offline demands is becoming increasingly risky and costly. UAV technology continues to mature and shows promising application prospects in the field of e-commerce logistics. By deploying drones and establishing offline collection demand offices, we can promptly gather the needs of groups such as the elderly with limited mobility and minors who cannot go out independently. This approach also reduces the risk of infection associated with manual collection, effectively avoids traffic congestion and poor road conditions, lowers the cost of logistics services during information collection and feedback, and significantly enhances service efficiency [24].

#### 4.1.4 Application of RFID technology

As one of RFID technologies, Electronic Article Surveillance (EAS) can tag each offline service demand order with its unique number and label [25]. Whenever a service demand is completed, the EAS tag can be deactivated, indicating that the demand has been satisfied. When an unsatisfied demand order passes through an EAS detection device, it can also be detected and uploaded to the Qbnb platform for re-service. The application of EAS technology can effectively ensure the traceability of demand orders and prevent them from being neglected or incomplete. In addition, RFID technology can be employed in Qbnb logistics control systems, with RFID readers scattered in designated areas and connected to the data management system. When a demand order passes through the reader, it automatically scans the information on the tag and inputs the data into the Qbnb platform for order storage, analysis, release, and logistics control.

#### 4.1.5 Integration with mainstream purchase and service e-commerce platforms

**Fig 10:**
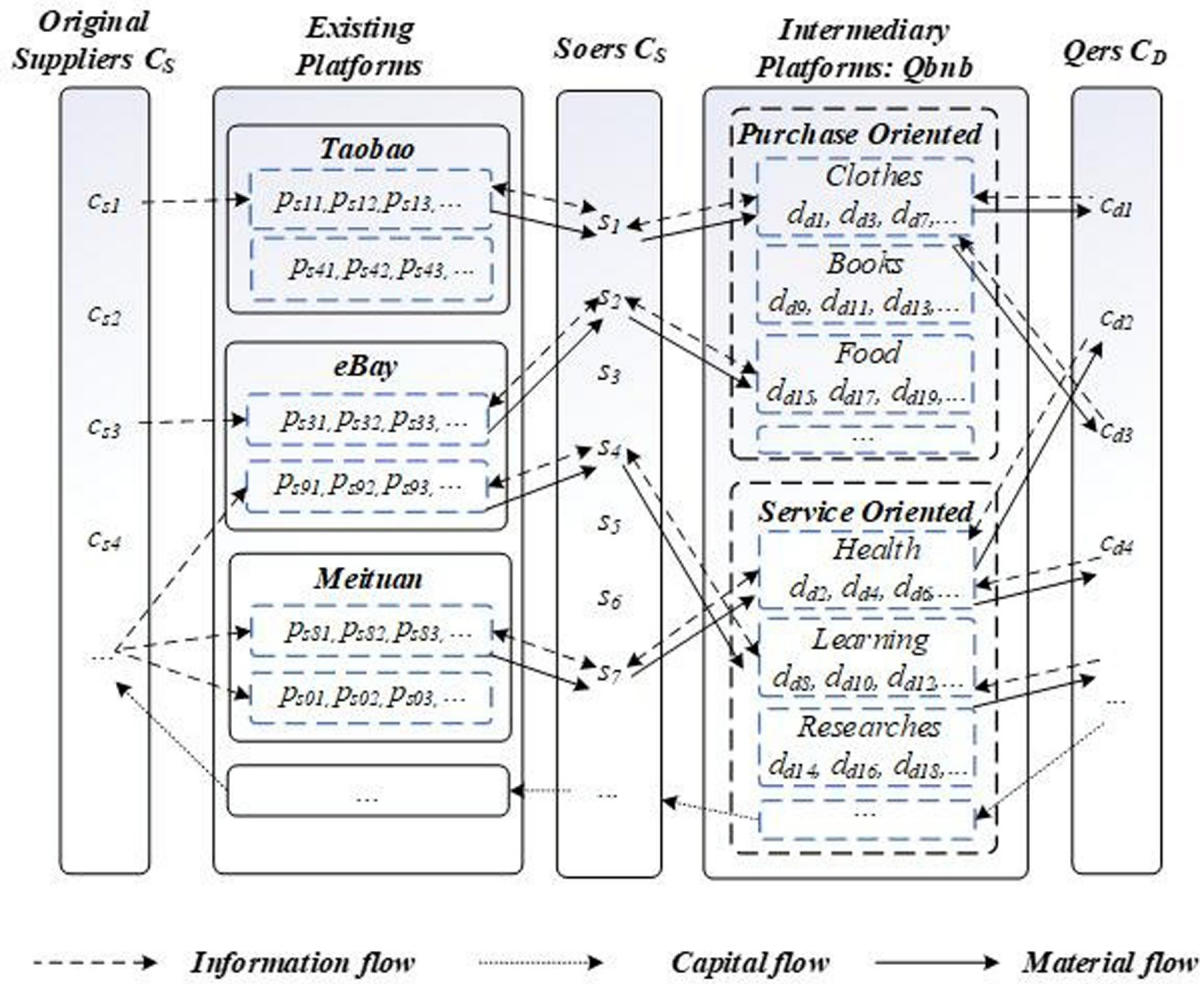
The integration of "Qbnb" platform and e-commerce platform in terms of information flow, material flow and capital flow.

The key to the C2B2C2B2C-based “Qbnb” e-commerce service mode is to connect the "Qbnb" intermediary platform and "Soers" (C_S_ side) with the existing e-commerce purchase/service platform and service demanders. Under the unified management of the "Qbnb" intermediary platform, Soers search for and create solutions from Taobao, eBay, and other mainstream e-commerce platforms or from the real world through order-grabbing. They then satisfy the service demands from Qers. In the “Qbnb” mode, various platforms, clients, administrators, etc. assume distinct roles in the service process, and three levels of mutual transformation and integration of information flow, material flow, and capital flow are achieved through the “Qbnb” platform [26], as illustrated in Fig 10.

The Information flow occurs as follows: Orders are initially passed from Qers to the “Qbnb” platform, which then forwards them to Soers. Subsequently, Soers search for solutions that meet Qers’ demands from other e-commerce platforms. Once the solutions are found, the Qbnb platform reviews the information and provides feedback to Qers, completing the integration and information flow between the platforms.

The Material flow works as follows: In response to purchasing demand from Qers, Soers obtains the information about products and services that users want from other e-commerce platforms. Once the “Qbnb” platform reviews the information, Soers can proceed to directly purchase and send them to Qers, completing the integration and material flow between the platforms.

The Capital flow occurs as follows: When Qers raise a demand on the Qbnb Platform, they shall pay the corresponding purchasing or service fee, which the platform will hold temporarily. If the cost is not high, Soers directly serve or pay the purchase fee in advance and send the products to Qers. Following mutual evaluations, the advance fees are then returned to Soers by the platform. If the price of the purchased product is too high, Soers can apply to be reimbursed by the platform. This way, the capital flow of the Qbnb mode is realized.

The “Qbnb” mode integrates information flow, capital flow, and material flow between the “Qbnb” platform and other e-commerce platforms, forming a new multi-level supply chain structure that will greatly promote e-commerce prosperity. New technical cooperation and collaboration contracts will be established between platforms, fostering multidimensional cooperative relationships such as data sharing, information pushing, and price concessions, enabling Soers to search for service solutions more easily and conveniently while providing timely feedback to users. The C2B2C2B2C-based “Qbnb” e-commerce service mode contains enormous business opportunities, which will attract e-commerce giants such as Alibaba, JD.com, 58.com, eBay, etc. to intensify their efforts in the layout and cooperation of third-party online second-hand transactions [27]. Cooperation with e-commerce platforms can not only improve internal response speed and service quality to provide better and more convenient service to clients but also bring increased profits and profitability to e-commerce platforms, playing a mutually beneficial and win-win role.

### 4.2 “Qbnb” intermediary platform technical architecture

**Fig 11:**
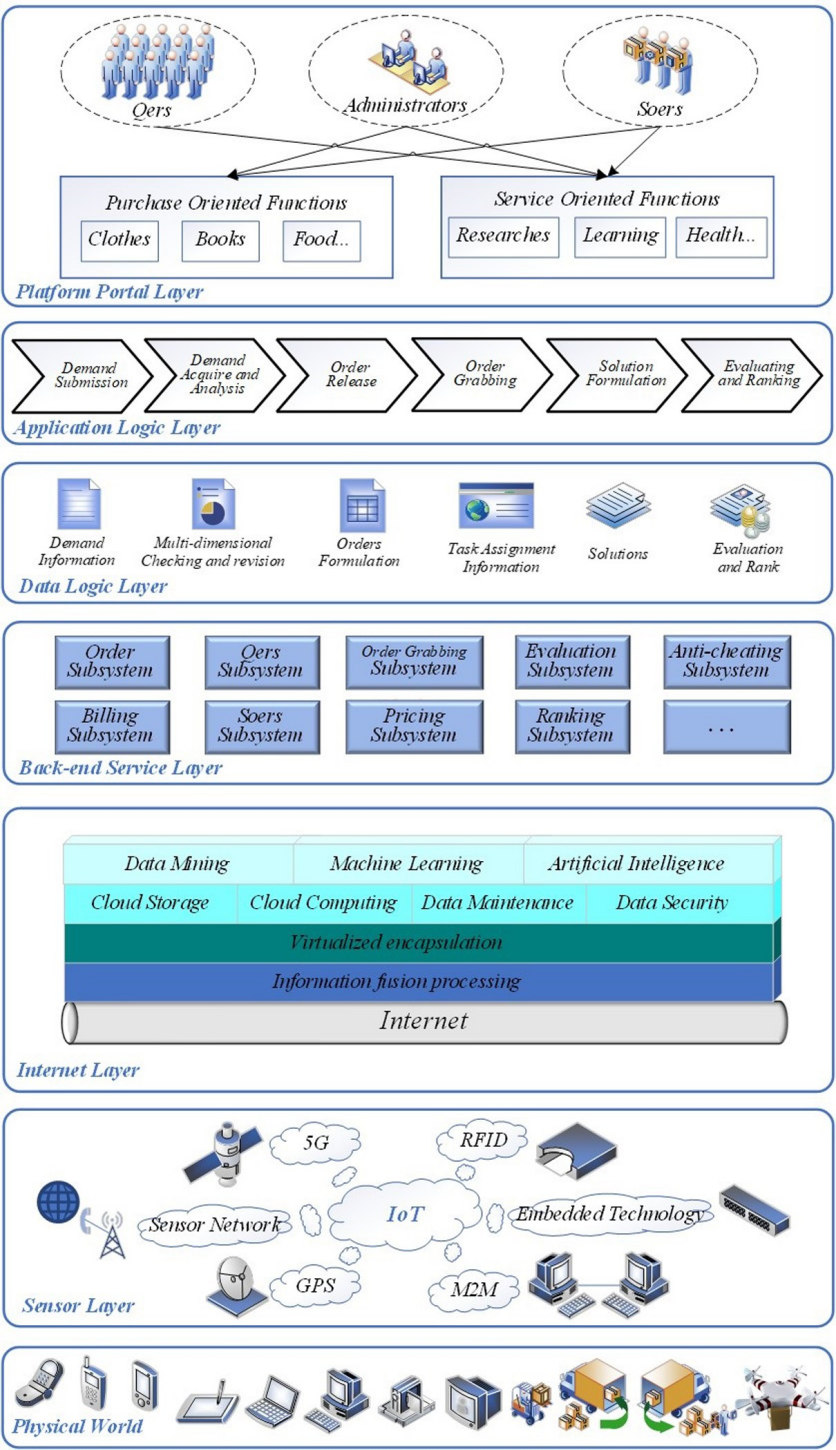
The architecture of the “Qbnb” platform.

The architecture of the “Qbnb” third-party demand release and integrated service response platform is depicted in Fig 11 and mainly comprises the following eight layers:

1. Physical world layer: This layer encompasses various mobile terminals, physical devices, and physical processes that require integration with the “Qbnb” platform to provide hardware support for information acquisition and interaction, IoT, and other functionalities. Key terminal devices include cell phones, tablets, laptops, desktop computers, cameras, monitors, and other electronic devices, along with logistics equipment and capabilities for transportation and loading during the distribution processes.
2. Sensor layer: This layer connects real-time state information of the physical world, such as Qers demand information, logistics distribution information, service process information, and other relevant information collected by terminal devices, to the Internet layer through new sensing technologies such as 5G network application technology, RFID technology, embedded technology, M2M data algorithm model technology, GPS satellite positioning technology, and sensor networks.
3. Internet layer: This layer encompasses the functionalities of information resource storage, computation, cleaning, integration, packaging, and maintenance, employing technologies such as information fusion, cloud computing, cloud storage, virtualization, data security, and others. Leveraging data mining, machine learning, artificial intelligence, and other technologies, the algorithm capabilities of “Qbnb” commercial big data analysis and computation will be realized, thereby providing data support and computational power for the seamless operation of the primary business functions on the platform.
4. Back-end service layer: This layer virtualizes and encapsulates the main business functions, algorithms, and logic into the common web service subsystem. Following the “Qbnb” mode outlined in Section 3, the platform is divided into nine major subsystems, such as the order subsystem, billing subsystem, Qers subsystem, Soers subsystem, order grabbing and distribution subsystem, pricing subsystem, evaluation subsystem, ranking subsystem, anti-cheating subsystem, and others. The platform’s business logic is implemented using modular and loosely coupled web services, which promote flexibility and scalability for future business activities.
5. Data logic layer: This layer provides support for structured, semi-structured, and unstructured data processing for the application logic layer, based on the “Qbnb” business operation logic. The data types handled by this layer include user demand information, multi-dimensional inspection and revision, solution formulation, task assignment, solution proposal, user evaluation, and Soers ranking. To store and access large amounts of structured and unstructured “Qbnb” business operation data in the layer, a high-performance, scalable, distributed database can be utilized. HBase is a suitable technology choice for this purpose, utilizing Hadoop HDFS as its file storage system and the Hadoop MapReduce algorithm to process the massive transaction data in HBase. Additionally, Zookeeper serves as a collaborative service to achieve efficient real-time access and processing of data.
6. Application logic layer: This layer implements the "Qbnb" operation mechanism, which involves demand submission, demand collection and analysis, demand allocation, order grabbing and distribution, solution proposal, and evaluation and ranking as proposed in Section 3. By utilizing interface-oriented programming and targeting specific business logic, the Qbnb platform can automatically invoke the web service from the service layer and access data from the data logic layer to create objects for seamless operations. This ensures the generation, access, processing, conversion, and storage of transaction data, as well as the specific realization of business functions.
7. Platform portal layer: The layer allows platform users, including Qers, Soers, and Administrators to access the portal via a web browser or a mobile terminal app. They can then choose between role registration or login and subsequently utilize a series of functional modules based on their user authorities. As per the findings from the Section 5 exploratory case studies, the platform incorporates both "purchasing-oriented" and "service-oriented" functional modules, which are further subdivided into various sub-functions based on the service types." The purchasing-oriented functions include buying items like clothes, books, and food, while the service-oriented functions consist of research, academics, health, and various other service-related functions. The subdivision of functional modules also determines the classification and attribution of users, especially Soers, which aids the platform in achieving more efficient and accurate matching of service providers with service demanders. This layer can utilize JSP (Java Server Pages) technology to dynamically generate responsive pages for the web front-end, integrate HTML and CSS technologies to typeset and layout the web pages, and combine JavaScript and Ajax technologies to perform asynchronous update operations on web pages. Additionally, it allows for other parallel technology implementation schemes, such as mini-program development tools.

## 5. Exploratory operation training

An exploratory operation, supported by the National Innovation and Entrepreneurship Training Program for University Students under the project titled “Qbnb: On-demand Service Mode (Project No. 202110555003X)”, has been conducted. Due to the team members being college faculty and students who needed to balance their studies and the program, the project has only undergone a trial operation for nearly 4 months, specifically from February 5 to June 2, 2022. Throughout this period, we collected and analyzed typical cases and related operational data to validate the innovation, feasibility, and application prospects of this mode. **The individual in this manuscript has given written informed consent (as outlined in PLOS consent form) to publish these case details.**

During the exploratory operation, team members played the roles of Administrators and Soers. Initially, the Qers were gradually attracted to put forward service demands by distributing leaflets. Due to limited funding, we temporarily did not develop a “Qbnb” platform and instead utilized WeChat groups and basic office software to imitate platform functions. The simplified “Qbnb” mode implementation process can be described as follows: Firstly, three Excel worksheets were used to replicate the order information databases, the Qers information database, and the Soers information database, respectively. Secondly, two online WeChat groups were established, including the Qers group and the Soers group, along with the setup of offline demand collection boxes. Thirdly, administrators collected and published service demands from/in Qers groups and offline collection boxes. Subsequently, Soers could grab orders from the Soers group and provide solutions to Qers through Administrators.

### 5.1 Typical case analysis

In this section, we present three representative cases of order processing in the exploratory operation stage. These cases demonstrate the advantages of diversified and demand-driven services in the “Qbnb” mode, proving its innovation and feasibility.

### Case 1: online commodity purchasing service (an example of money replacing time)

In pursuit of beauty, women often worry that the use of cosmetics will cause damage to their skin. Miss Xiong, one of the Qers, was suffering from acne and itchy skin due to prolonged use of cosmetics. She urgently wanted to buy a soothing repair essence to improve her skin. However, most of the soothing repair essences available on e-commerce platforms were expensive and had a wide range of options. Miss Xiong was under great pressure from her studies and work and could not afford to spend much time searching for products with suitable prices on e-commerce platforms.

**Fig 12:**
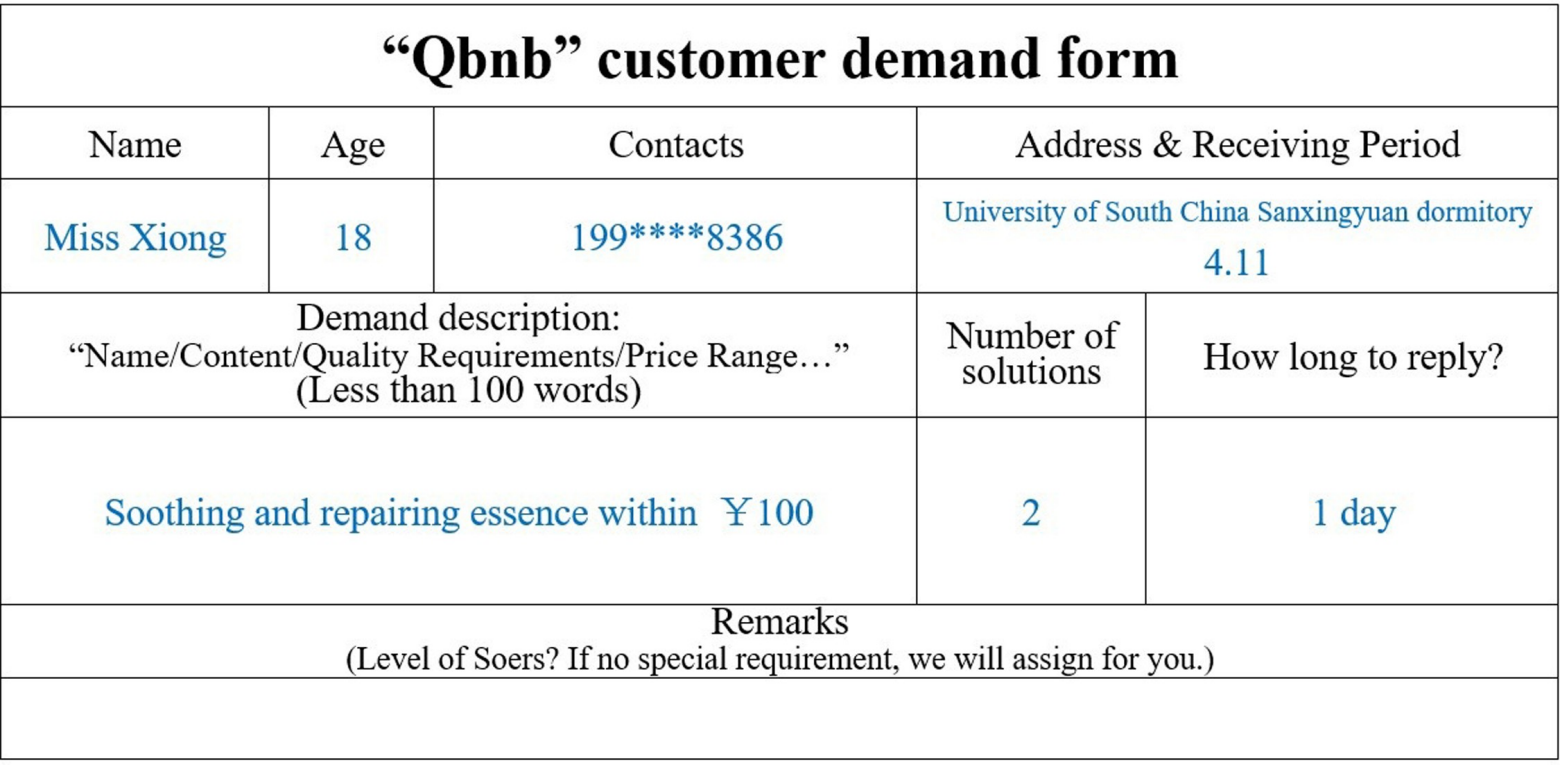
Qer Miss Xiong’s electronic demand form (translated).

**Fig 13:**
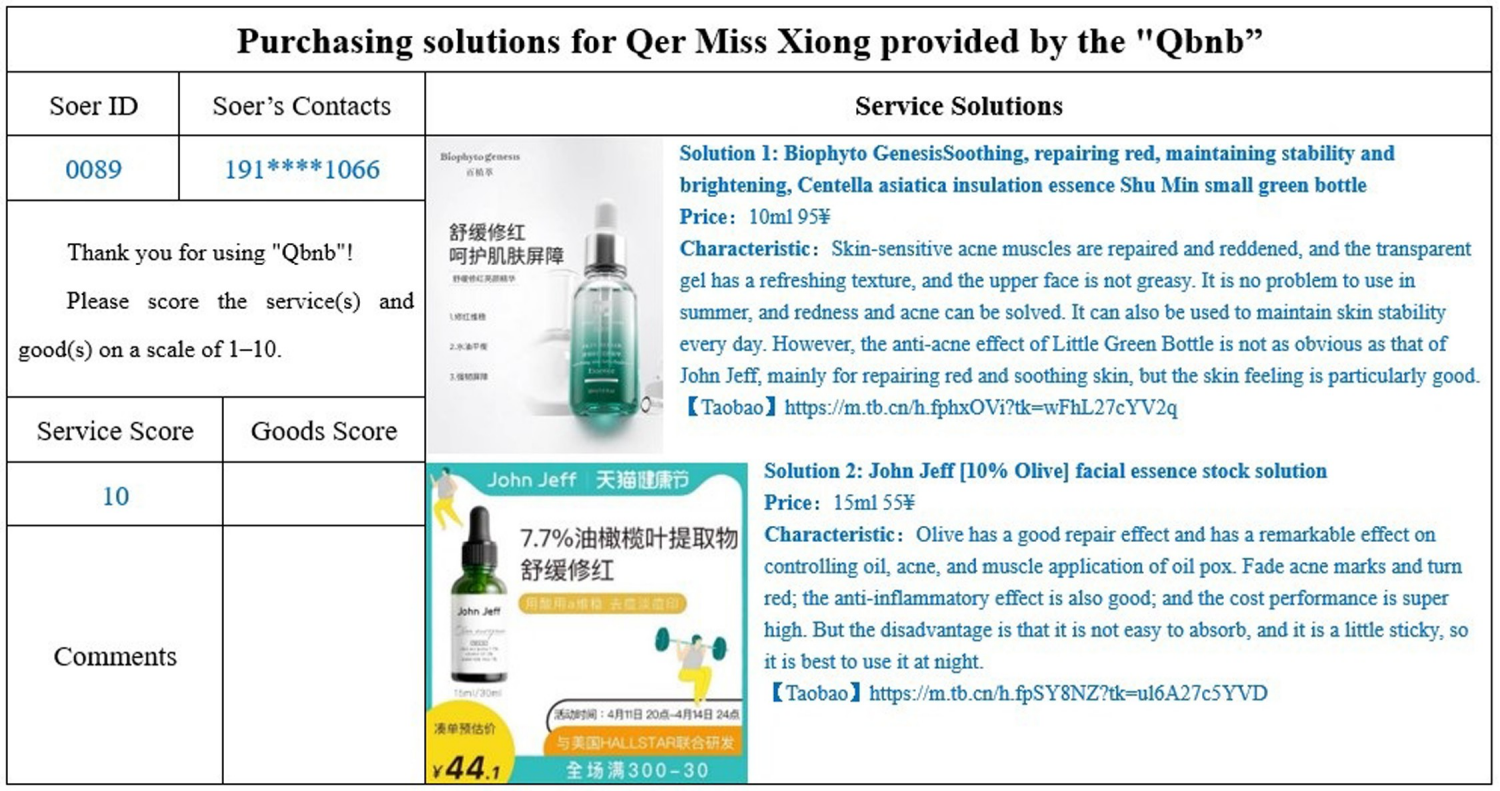
Purchasing solutions for Qer Miss Xiong provided by the “Qbnb” (translated).

Miss Xiong chose to fill out the order online and submitted her requirement in the "Qbnb" Qers Group (Fig 12), requesting Soers to provide two alternative purchasing solutions for less than one hundred yuan within one day. Yini Fang (Soer ID: 004), who has experience in purchasing cosmetics and essences, successfully took on the order from the Soers group and promptly searched Taobao, JD.com, and other e-commerce platforms based on Miss Xiong’s specific requirements. On the same day, Yini Fang provided the introduction and purchase proposal for the essence that met Miss Xiong’s demands, which was then sent back to the administrator for formal review (Fig 13). Subsequently, the administrator forwarded the purchase proposal to Miss Xiong for a final decision. Miss Xiong ultimately accepted Soer’s proposal, paid the “Qbnb” 25 RMB service fee through WeChat payment, and gave a full rating for both the service and the product in the evaluation section.

In this case, Miss Xiong’s urgent demand was met in a timely manner through “Qbnb”, allowing her to avoid the burdensome process of product selection and enjoy professional and high-quality purchasing services with very minimum time and effort. At the same time, “Qbnb” and Soer also received satisfactory feedback and the corresponding fee (25 RMB) from users, creating a win-win situation for all three parties. This also achieved the on-demand service goal of enabling time-poor e-commerce groups to substitute their time with money.

### Case 2: offline decoration company search service (an example of money replacing ability)

Grandma Peng, who is nearly seventy years old, wanted to decorate her new apartment simply. However, being unable to go out and look for a suitable decoration company due to her age, she was also unfamiliar with using the Internet for this purpose.

**Fig 14:**
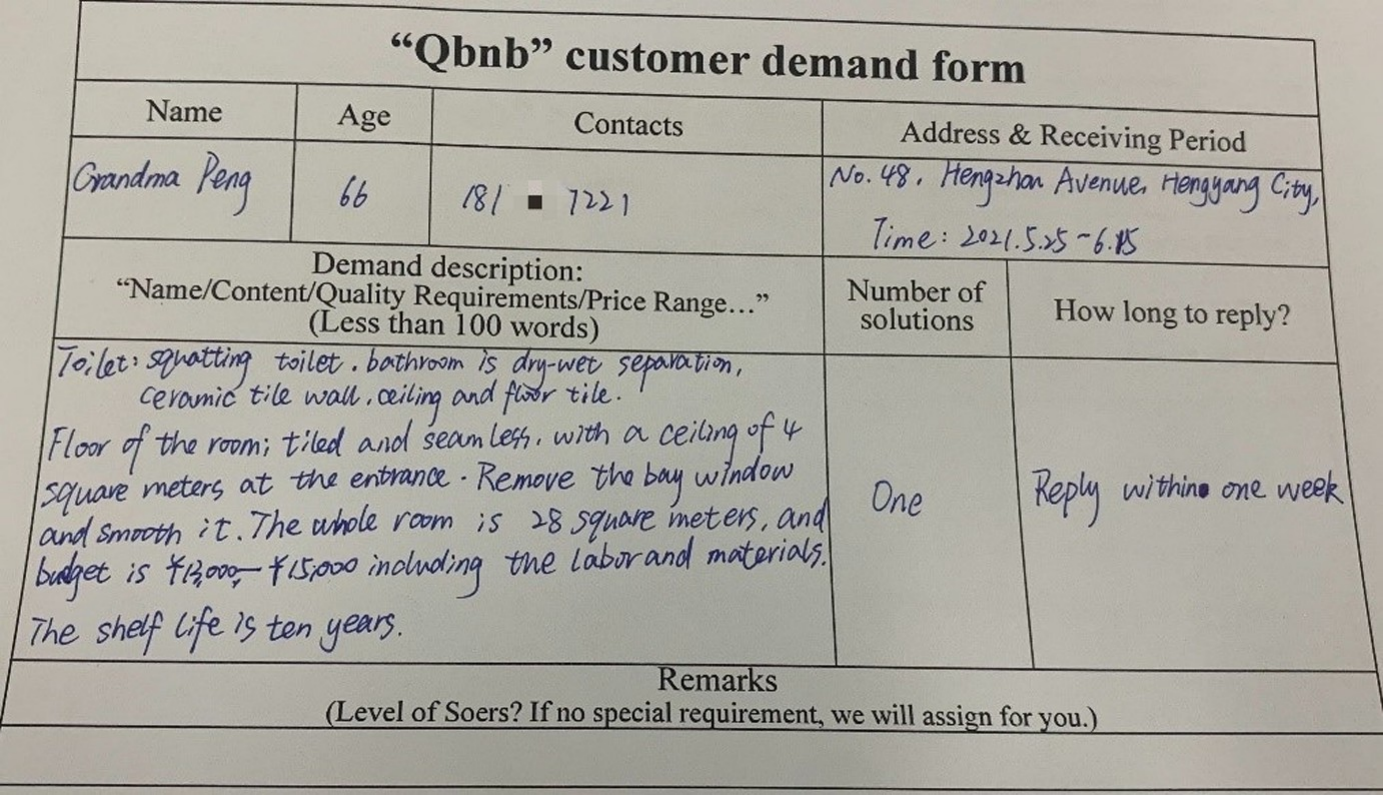
Qer Grandma Peng’s paper service demand form (translated).

**Fig 15:**
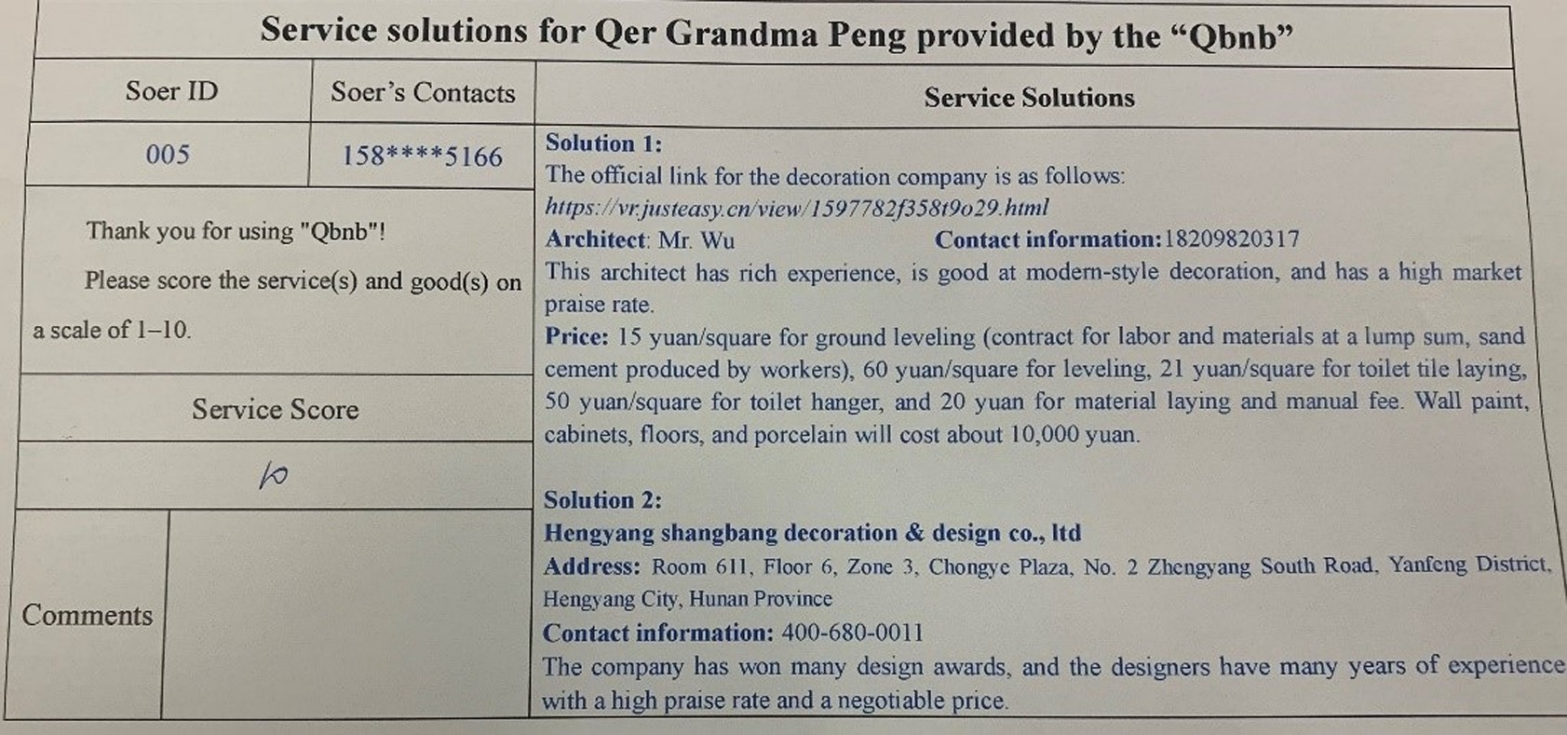
Service solution for Qer Grandma Peng provided by the “Qbnb” (translated).

Grandma Peng filled out the paper demand form, requesting Qbnb to recommend two decoration companies that meet the requirements within a week (refer to Fig 14), and submitted it to the “Qbnb” offline demand collection box in the community. Each day, the “Qbnb” team gathers offline service demands and forwards them to the administrator in a unified manner. Once reviewed, the collected demands are published as an order in the Soers group. Kangxin Yu (Soer ID: 005), who has extensive experience in decoration, successfully secured this order and promptly searched the Internet using her own expertise. She recommended two decoration companies to Qer Peng and provided the template display link of the companies’ products for decision-making reference. After a quick review of the recommendations (Fig 15), the administrator immediately gave feedback to Grandma Peng regarding her decision-making. Grandma Peng adopted the plan provided by Soer and contacted one of the decoration companies. Afterwards, Grandma Peng paid 110 RMB to “Qbnb” for the service through WeChat payment and gave full marks for both the service and the outcome in the evaluation section.

In this case, Qer Peng could receive satisfactory service without leaving home, and the “Qbnb” team and Soer also obtained a considerable income and high score evaluation, leading to a win-win situation for all three parties. This proves that even vulnerable groups of elderly e-commerce consumers, who have no online consumption ability at all, can easily enjoy high-quality online business resources and services under the “Qbnb” on-demand service mode, just like young people.

### Case 3: online intractable diseases or problems solving service (an example of money replacing ability and time at the same time)

**Fig 16:**
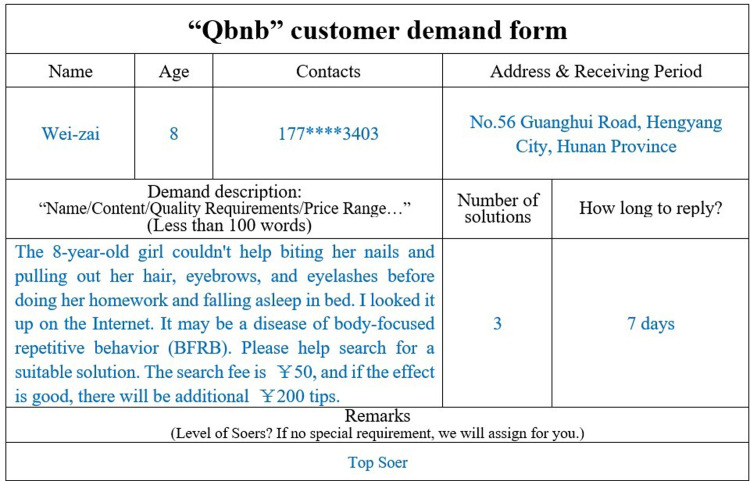
Qer Wei-zai’s electronic demand form (translated).

Wei-zai, an 8-year-old girl, suffers from a "hair-pulling and nail-biting" disorder. She often uncontrollably and unconsciously pulled out her eyebrows, hair, and gnawed off her fingernails when she concentrated on reading and studying, without feeling any pain or itch. Her parents were very worried about this because there was no way to always keep an eye on her, especially when she went to school during the day. At the same time, they were concerned that taking her to the doctor might cause stress and burden on their child’s psyche. So, her dad chose to fill out the order form online and present his request in the "Qbnb" Qers group (Fig 16), asking Soers for three referable solutions within three days and expressing a willingness to pay 50 to 200 RMB to the “Qbnb” team for this service.

**Fig 17:**
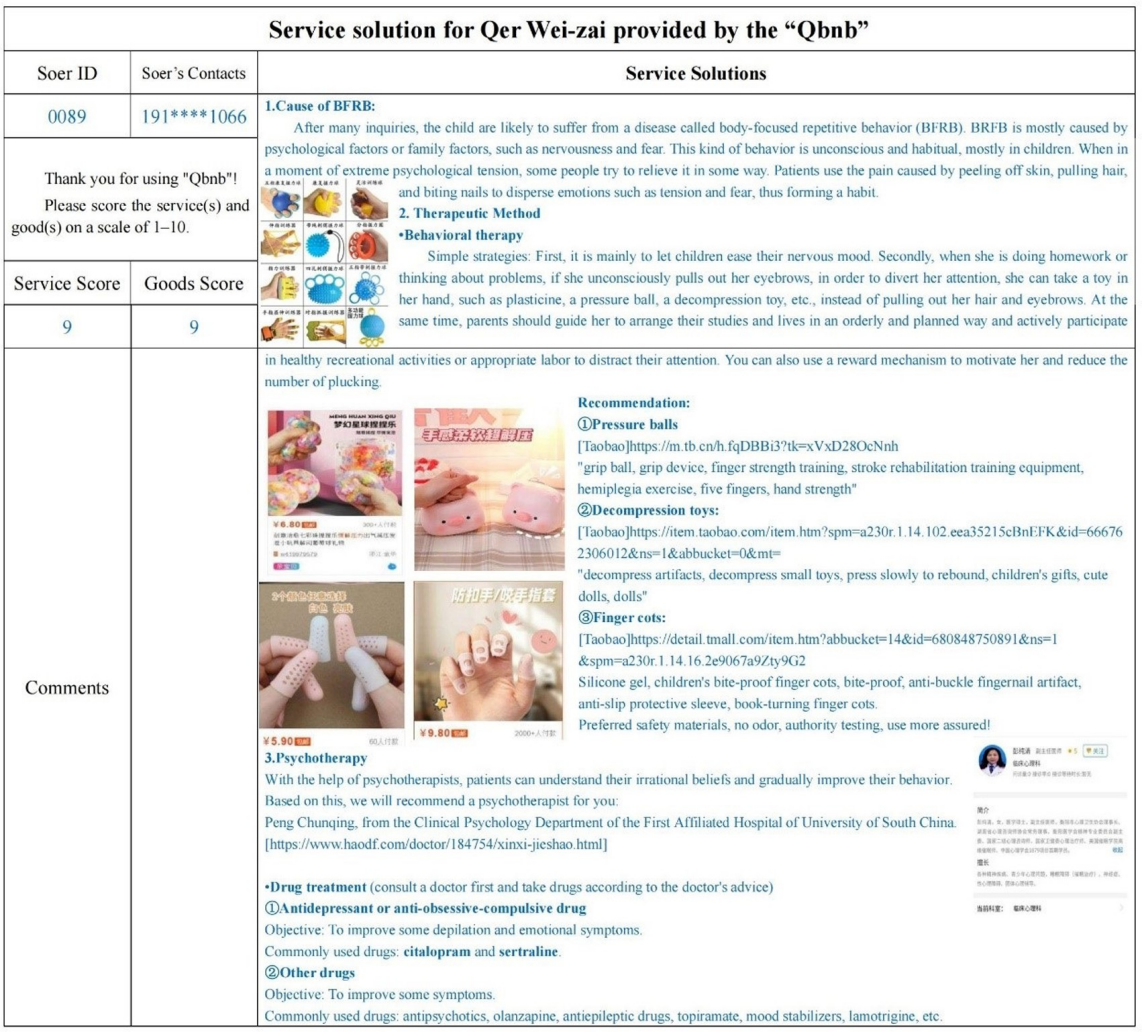
Service solution for Qer Wei-zai provided by the “Qbnb” (translated).

"Kangxin Yu (Soer ID: 005)", who is good at searching for solutions to intractable diseases or problems, took on the case. Following online research and offline consultation, she identified that Wei-zai’s symptoms were indicative of Body-Focused Repetitive Behavior (BFRB). Additionally, Soer provided the causes of BFRB and presented three detailed treatment options, which included behavioral therapy, psychotherapy, and drug therapy. Behavioral therapy includes using stress-reducing toys, such as a decompression ball, to distract children, or wearing finger cots to prevent her from "hair-pulling." In the receipt, Soer provided an introduction and purchase links for related auxiliary products of behavioral therapy. Additionally, they recommended local authoritative doctors for psychotherapy and commonly used drugs for drug treatment (Fig 17). Finally, Qer implemented a behavioral therapy program that effectively suppressed the child’s pulling hair and biting nails behaviors. Afterward, Qer made the payment to the "Qbnb" team through WeChat and gave them a top score in the evaluation section for their service.

There are many intractable diseases or problems like this one in our high-pressure modern society. Qers often do not have sufficient time or ability to effectively solve such issues. However, with the assistance of the "Qbnb" team, the intractable diseases that had caused Qer headaches for a long time were perfectly solved with only a small amount of money. On the other hand, with the support of the platform and search capabilities, the "Qbnb" team and Soer successfully completed the service, quickly earning the service fee and receiving praise, resulting in a win-win situation for all three parties. The case demonstrates that the "Qbnb" mode can not only offer regular online and offline commodity purchasing and consulting services based on the demands of Qers but also assist in finding satisfactory solutions for their special needs, such as intractable diseases, problems, and life difficulties. This realization enables the optimization of social idle time, abilities, and financial resources.

The three typical cases above illustrate that under the demand-driven “Qbnb” service mode, Qers can submit various types of service demands either online or offline. Whether it is a special case, such as a commodity purchase, consulting service, or intractable diseases or problems, the “Qbnb” team and Soers can offer services on demand as long as Qers provide formal or informal specific constraints and price ranges.

### 5.2 Exploratory operations analysis

#### (1) Analysis of orders and service revenue during exploratory operation

1. During the exploratory operation, varied types of Qbnb orders were completed, including 47 online and 109 offline orders (order details are available in Appendix A). The service revenue amounted to 3,027.5 yuan, generated by one administrator and five Soers participants (504.5 yuan per person). The six part-time participants were all college students. Excluding daily study and rest time, each person participated in the “Qbnb” service for less than 1 hour per day, i.e., \6.6 per hour per capita. Considering the short exploratory operation time and the low service price in the initial stage to attract Qers, it is a nice part-time income option.

**Fig 18:**
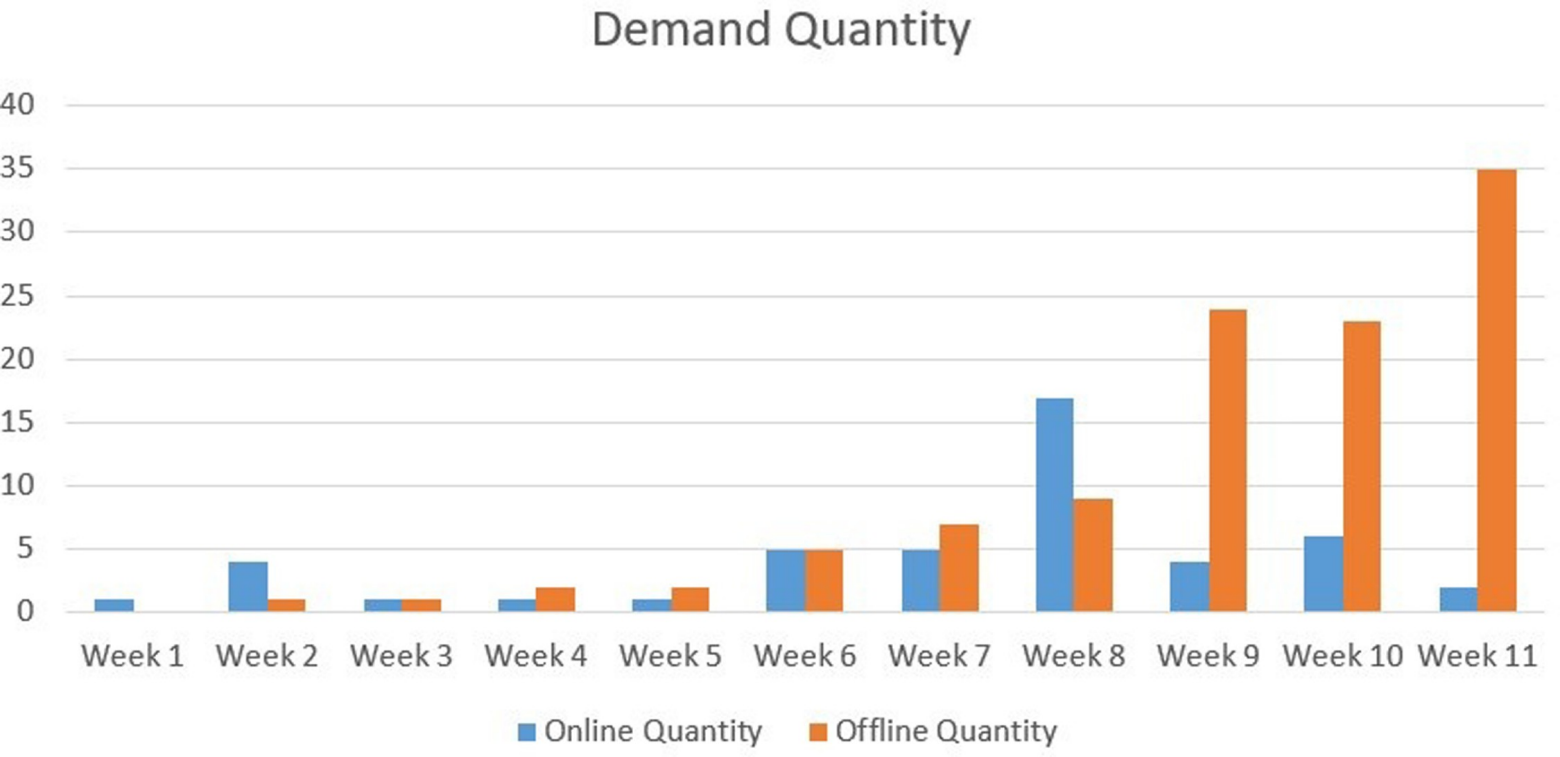
Weekly demand quantity during the trial operation of the “Qbnb”.

**Fig 19:**
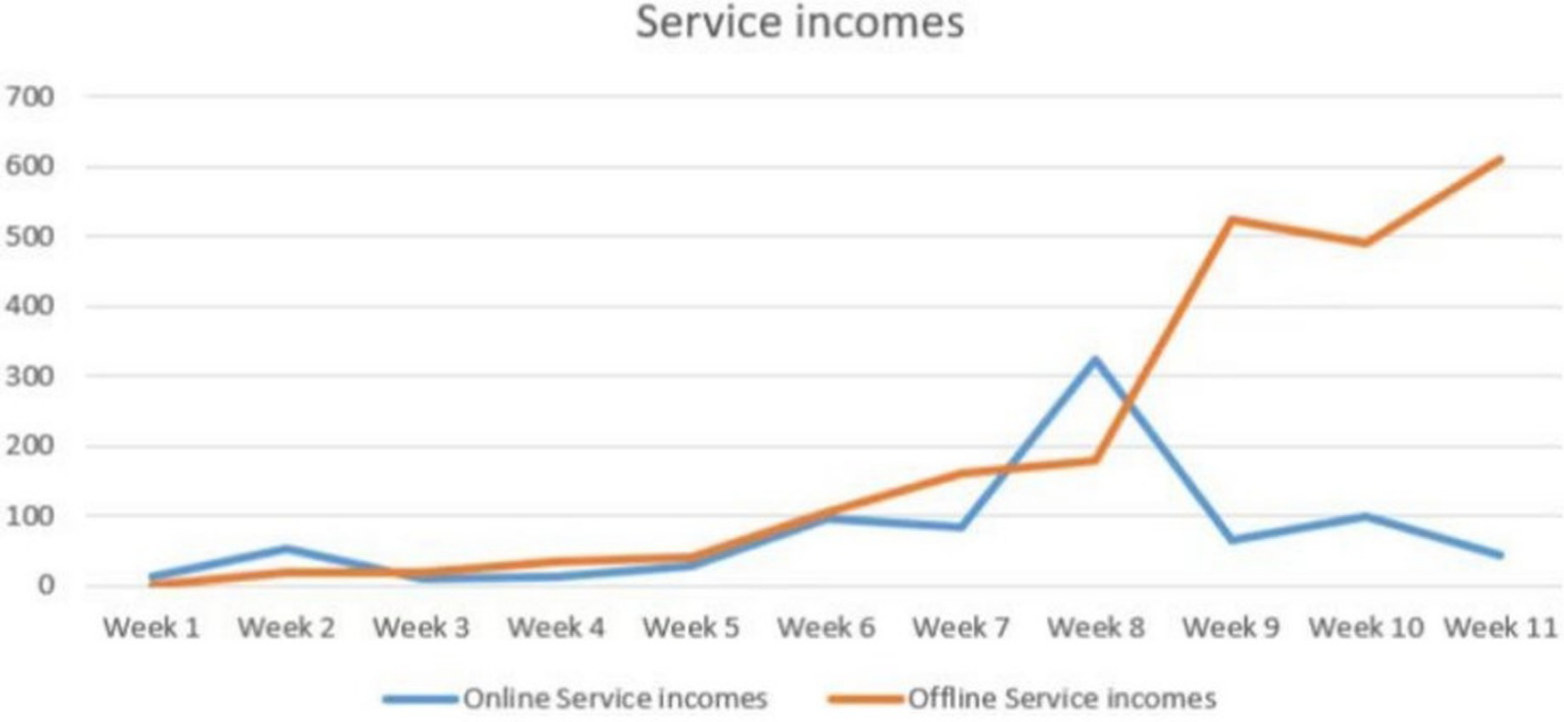
Weekly turnover trend during the trial operation of the “Qbnb”.

As shown in Figs 18 and 19, the number of online orders and service revenue tends to increase in the first eight weeks, peaking in the eighth week, and beginning to decline in the last three weeks. The decline in online orders and service revenue during the final three weeks can be attributed to finals week, as many student customers are focused on exam preparation. The overall trend for offline orders is steadily increasing. It is evident that “Qbnb” is progressively improving its collection of offline demand, effectively attracting users to place orders. Undoubtedly, in the later development process of “Qbnb”, online and offline parallelism will be the way to go. At present, both sides have high activity and development potential. Given the large number of individuals with online consumption ability in society, it is foreseeable that the “Qbnb" platform’s future development and application will significantly boost online service activity. With enhanced market awareness, the “Qbnb” mode is poised to serve more Qers and generate considerable income for more Soers.

#### (2) Analysis of Qers age distribution and service types during the exploratory operation

**Fig 20:**
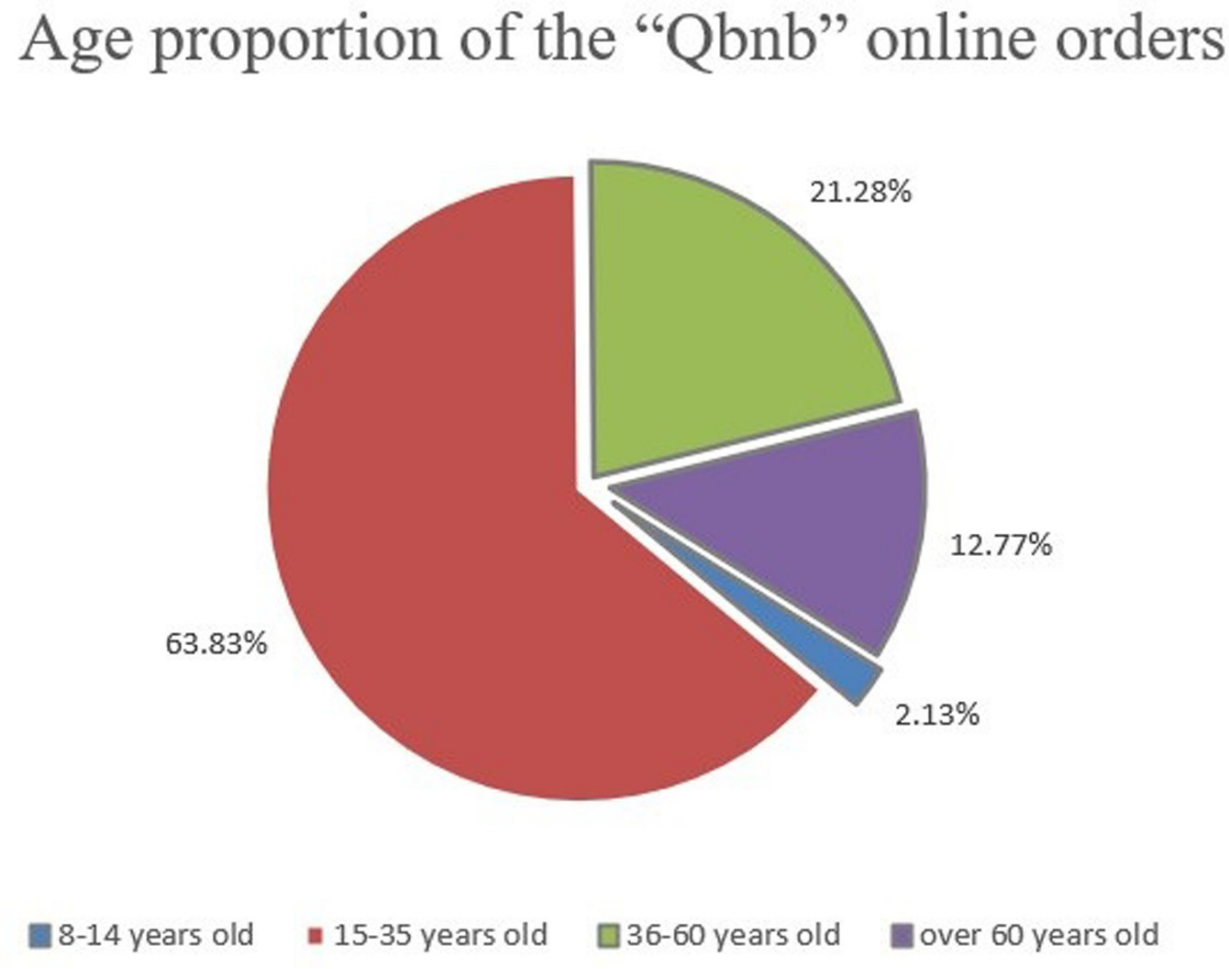
Age proportion of the “Qbnb” online orders.

**Fig 21:**
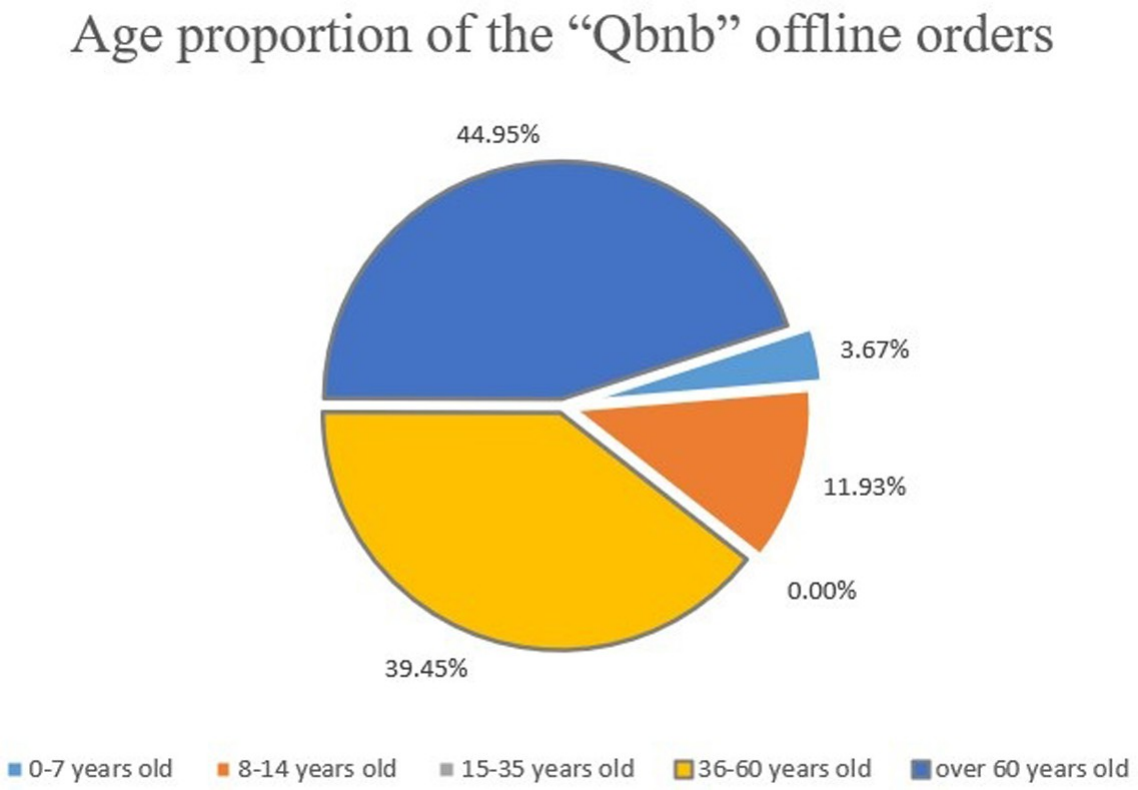
Age proportion of the “Qbnb” offline orders.

1. During the exploratory operation, online orders were dominated by young people aged 15–35 and middle-aged people aged 36–60, accounting for 63.83% and 21.28%, respectively (Fig 20). These Qers are familiar with the online consumption process, and most of them choose the “Qbnb” platform for efficient purchasing of goods or problem-solving services due to the high time cost of online shopping or a lack of ability and experience in online search. Nearly half of the offline orders come from the group of senior Qers over 60 years old (Fig 21). They are not familiar with online consumption. Especially during pandemics, when goods are in urgent need due to regional shortages or when they cannot be purchased independently because of mobility difficulties, they can only submit their demands offline. They then ask the “Qbnb” team to provide purchasing services or other assistance.

**Fig 22:**
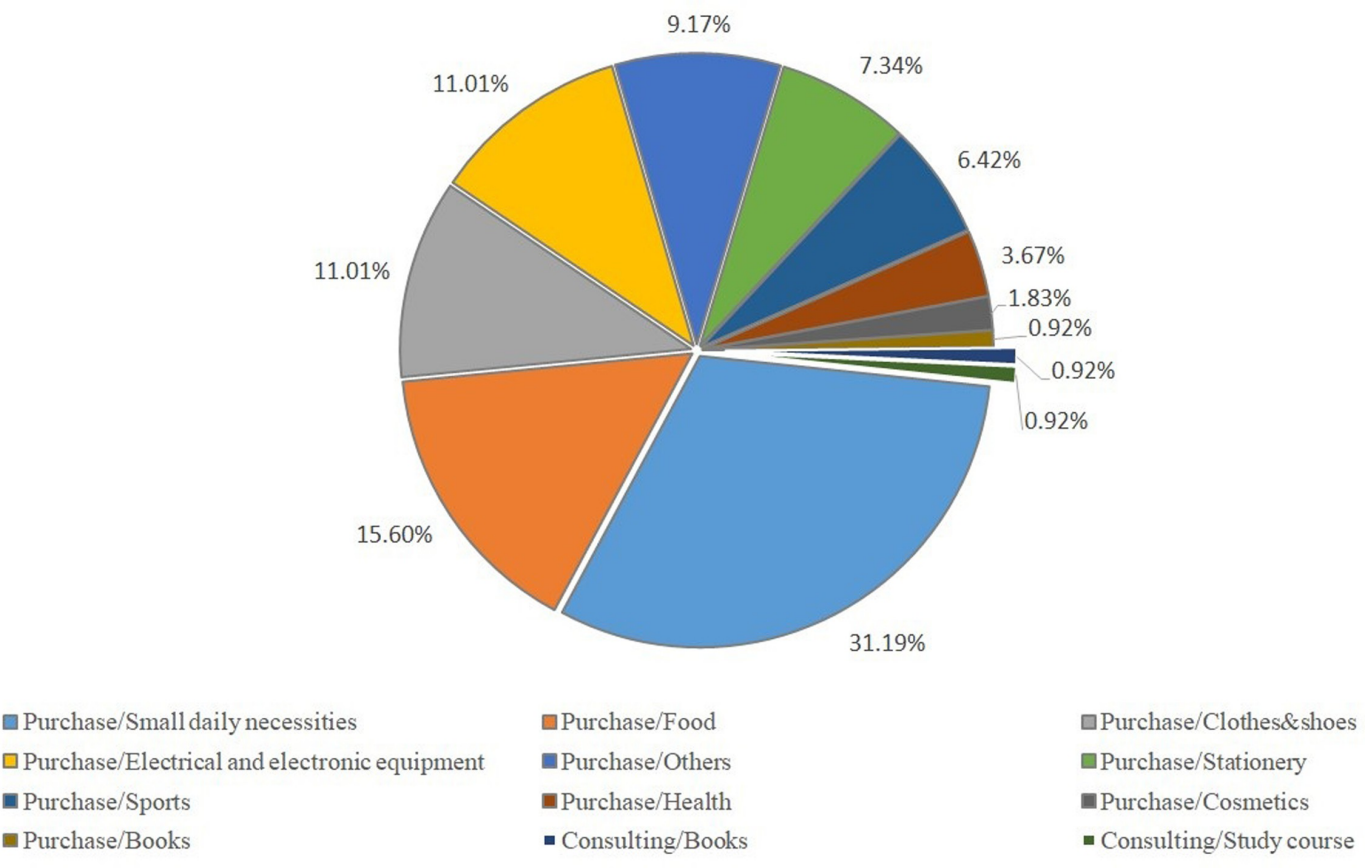
Types and proportion of offline service orders during exploratory operation.

**Fig 23:**
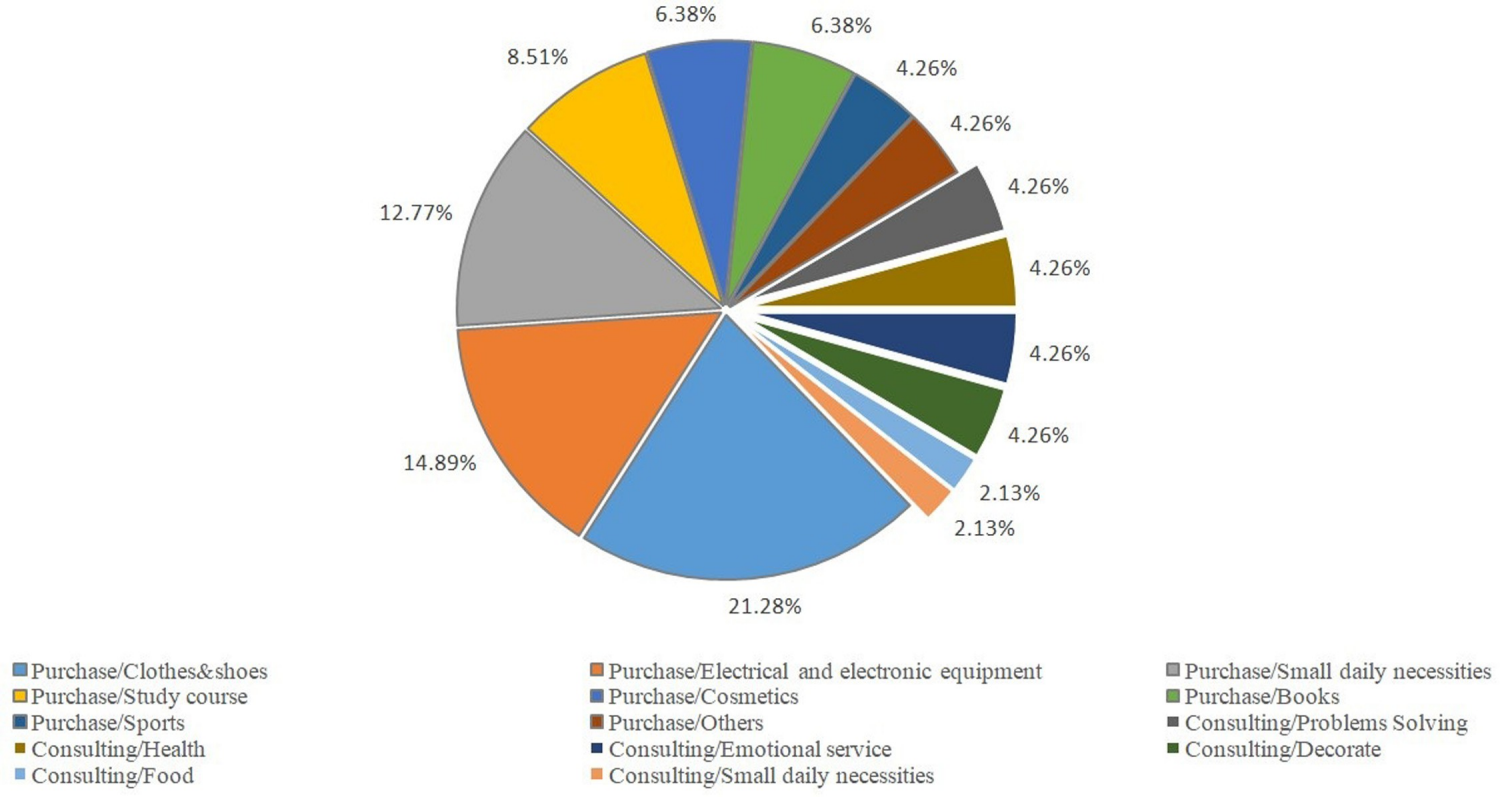
Types and proportion of online service orders during exploratory operation.

During the exploratory operation, there are two major categories of orders: purchasing services and consulting services. For offline services (Fig 22), entity purchasing services account for a significantly higher proportion compared to consulting services. The top three orders for small daily necessities—food, clothes, and shoes—comprise 31.19%, 15.6%, and 11.01% of the total, respectively. As most offline clients are elderly people who lack the ability to shop online, the "Qbnb" team purchases their daily necessities directly and arranges home delivery through third parties. The steadily increasing offline orders (Fig 19) demonstrate that the gradual recognition of the "Qbnb" offline service mode within its operational area by this demographic. The online services mainly consist of purchases, with clothes and shoes, electrical equipment, and small daily necessities being the top three, accounting for 21.28%, 14.89%, and 12,77% of orders, respectively (Fig 23). It shows that mainstream market demand is still focused on commodity purchasing services, both online and offline. In addition, there are 21.3% of orders for consulting services online, which include addressing intractable diseases or problems, answering health questions, providing emotional services, providing decoration services, and more. Due to people’s dependence and inertia, an increasing number of online clients are now inclined towards receiving one-stop shopping services and opting for home delivery. In the foreseeable future, when artificial intelligence cannot entirely replace humans (Soers) for certain Qers’ service requirements, the "Qbnb" might serve as an indispensable intermediate node that neither traditional e-commerce service platforms nor Qers (end customers) can bypass.

## 6. Conclusions and future work

### 6.1 Conclusions

C2C, B2C, and B2B e-commerce platforms like Taobao, eBay, Amazon, and Alibaba offer vast amounts of structured product information, allowing the demand-side to choose from a supply-side perspective. However, these traditional modes lack a personalized description and explanation mechanism for e-commerce services. As a result, e-commerce vulnerable groups, facing challenges such as high time costs, limited online shopping ability, and unclear personalized needs, may invest a significant amount of time but find it difficult to be adequately catered to. To address this problem, the "C2B2C2B2C E-Commerce Mode" is proposed as an innovative solution for integrated on-demand services. After conducting exploratory operations, the relative advantages and contributions of this mode are verified. Five conclusions are drawn, as described as follows:

1. On-demand service: The “Qbnb” service mode is driven by the demands of Qers. Through this mode, Soers service providers can capture orders and provide tailored service solutions for Qers based on their specific demands. On one hand, the “Qbnb” significantly improves the satisfaction of consumers’ personalized service demands compared to the traditional supply-side-driven e-commerce service mode. On the other hand, the "Qbnb" team and Soers’ manual analysis enable accurate understanding and efficient handling of unstructured and ambiguous service demands that cannot be mechanistically explained by traditional e-commerce platforms, thereby enhancing on-demand service capabilities.
2. Integrated services: The "Qbnb" mode is designed to connect the service provider group (Soers) with "time and capacity" and a service demand group (Qers) with "demand and capital" on the "Qbnb" intermediary platform, facilitating the integration and matching of idle surplus service capacity within society with the service demand of e-commerce vulnerable groups. This innovative "Qbnb" integration mode allows anyone to become a Qer or Soer, significantly expanding service demands and capabilities. It also enables higher service efficiency, fosters a broader service market, and creates more job opportunities.
3. Stronger service capability: By establishing mechamisms such as "Soers Training", "Order Grabbing", "Mutual Evaluation", Qbnb can create an experienced, professional, and systematic Soer service team. This team will efficiently search for and offer service solutions, significantly saving consumers’ time and cost compared to traditional e-commerce modes. Moreover, it will enable Qers to access on-demand services more efficiently.
4. Broader service consumption market: The ’Qbnb’ mode creates a broader service consumption market by facilitating communication, collection, screening, classification, understanding, publishing, and management of vague, unstructured service demands. This approach addresses more potential market demands that cannot be expressed and explained under the traditional e-commerce mode driven by the supply side (e.g., consultation on intractable diseases or problems, offline help for the elderly, academic consultation, etc.), leading to their satisfaction and the emergence of a new and broader consumption market.
5. Create more jobs: Under the Qbnb model, Soers can provide on-demand services for Qers while also receiving corresponding service remuneration, enabling them to become full-time or part-time staff and thereby creating more jobs. The “Qbnb” will not only stimulate the consumer market but also generate more job opportunities. In today’s post-pandemic era of networked offices, “Qbnb” allows more people to find flexible job opportunities from the comfort of their homes and achieve a full harvest.

### 6.2 Limitation and future work

Due to project funding and manpower constraints, we have only designed the basic operational process and the prototype of the platform technology architecture for the “Qbnb” mode. We conducted an exploratory simulation operation based on a WeChat group to initially verify the advantages of the mode and its broad future development prospects. In the next step, our research will focus on the development, implementation, and continuous improvement of the “Qbnb” platform. We will strive to carry out the actual operation and further explore the consumers’ behavior rules and business operation mechanisms under the “Qbnb” mode.

## Supporting information

S1 Data Data supporting information. (RAR)
